# Boiogito Ameliorates Inflammation-Associated Adipocyte Dysfunction and Restores Adipogenesis in Association with Suppression of NF-κB Signaling

**DOI:** 10.3390/cimb48070693

**Published:** 2026-07-08

**Authors:** Yi Luo, Ailing Hu, Jingya Lu, Wenshu Yuan, Yu Tan, Takuji Yamaguchi, Zenji Kawakami, Yasushi Ikarashi, Yoshinao Harada, Hiroyuki Kobayashi

**Affiliations:** Department of Personalized Kampo Medicine, Juntendo University Graduate School of Medicine, Tokyo 113-8421, Japan; ailing@juntendo.ac.jp (A.H.); w.yuan.tg@juntendo.ac.jp (W.Y.); y.tan.re@juntendo.ac.jp (Y.T.); tkyamagu@juntendo.ac.jp (T.Y.); z.kawakami.sj@juntendo.ac.jp (Z.K.); y.ikarashi.zi@juntendo.ac.jp (Y.I.); yo-harad@juntendo.ac.jp (Y.H.); koba@juntendo.ac.jp (H.K.)

**Keywords:** Boiogito, TNF-α, 3T3-L1, adipocyte differentiation, inflammation, NF-κB

## Abstract

Boiogito (BOT), a traditional Kampo herbal medicine, has been reported to exhibit anti-inflammatory and anti-obesity properties. However, its potential role in protecting adipocyte function under inflammatory conditions at different stages of adipocyte development remains unclear. This study investigated the effects of BOT on adipogenesis and tumor necrosis factor-α (TNF-α)-induced inflammatory responses in differentiating and mature 3T3-L1 adipocytes. In this study, 3T3-L1 preadipocytes were induced to differentiate and exposed to TNF-α in the presence or absence of BOT during differentiation or after full adipocyte maturation. Lipid accumulation was assessed by Oil Red O staining, while adipokine secretion and inflammatory cytokine production were evaluated by ELISA. The expression of adipogenic markers and inflammatory signaling molecules was analyzed using quantitative PCR and Western blotting. TNF-α significantly inhibited the expression of adipogenesis-related factors at the transcriptional level in adipocytes, reduced lipid accumulation and adiponectin expression, and enhanced inflammatory cytokine production. BOT treatment dose-dependently attenuated these effects, restoring adipogenic capacity and suppressing inflammatory responses in both differentiating and mature adipocytes. Mechanistically, BOT reduced TNF-α-induced activation of the NF-κB pathway, as evidenced by decreased phosphorylation of NF-κB p65 and IκB. These findings demonstrate that BOT preserves adipocyte function and mitigates inflammation-associated adipocyte dysfunction throughout adipocyte development. The protective effects of BOT may contribute to the regulation of obesity-associated metabolic inflammation, partly through modulation of NF-κB signaling.

## 1. Introduction

In recent years, obesity has become a major global health concern, being closely associated with a range of metabolic diseases, including insulin resistance, type 2 diabetes, and cardiovascular disease [1,2]. Obesity is associated with chronic low-grade inflammation. This persistent inflammatory state, often called “metaflammation”, acts as a link between obesity and metabolic dysfunction [3]. Among the tissues involved in metabolic regulation, adipose tissue plays a central role in the development and progression of obesity-associated diseases.

Adipose tissue is considered an active endocrine organ rather than a passive reservoir for energy storage, playing a central role in systemic metabolic regulation [4] by secreting various adipokines, cytokines, and chemokines [5,6]. Under energy excess, adipose tissue expands by increasing adipocyte size and number. Although adipocyte hyperplasia is a relatively favorable adaptive response, excessive hypertrophy is more often associated with adipose tissue dysfunction, characterized by hypoxia, fibrosis, and increased immune cell infiltration [7,8]. These alterations promote a pro-inflammatory microenvironment, which aggravates metabolic dysfunction.

Adipocyte differentiation (adipogenesis) is a highly coordinated process regulated by multiple transcription factors, among which peroxisome proliferator-activated receptor γ (PPARγ) and CCAAT/enhancer-binding protein α (C/EBPα) play central roles [9]. Its regulation is crucial for maintaining normal adipose tissue function and metabolic homeostasis. However, this process is highly sensitive to inflammatory signals. Pro-inflammatory cytokines, including tumor necrosis factor-α (TNF-α), can interfere with the adipogenic transcriptional program, leading to impaired adipocyte differentiation and increased insulin resistance [10,11]. Simultaneously, impaired adipogenesis can aggravate inflammation by promoting adipocyte hypertrophy and macrophage recruitment [12]. These findings suggest a bidirectional interaction between adipogenesis and inflammatory signaling in adipose tissue.

Among the inflammatory pathways involved in obesity-associated adipose dysfunction, the nuclear factor-κB (NF-κB) pathway is considered a major mediator of inflammatory responses. Under stimulation by cytokines such as TNF-α, NF-κB signaling enhances the expression of pro-inflammatory mediators, including interleukin-6 (IL-6) and monocyte chemoattractant protein-1 (MCP-1), thereby promoting immune cell infiltration and leading to systemic metabolic disorders [13,14]. Given the close interplay between inflammatory signaling and adipocyte function, targeting this axis holds promise for the treatment of obesity-associated metabolic diseases.

Natural products and traditional herbal medicines have the potential to modulate metabolic disorders. Numerous plant-derived compounds have been shown to affect adipogenesis and inflammation, largely by regulation of transcription factors and signaling pathways [15,16,17]. However, many previous studies have employed simplified experimental models that focus either on adipocyte differentiation or inflammatory responses alone, while overlooking the distinct physiological characteristics of differentiating and mature adipocytes. As adipocyte responses to inflammatory stress may vary depending on differentiation status, the context-dependent regulatory mechanisms underlying adipocyte inflammation and dysfunction remain insufficiently understood.

Boiogito (BOT) is a traditional Kampo medicine composed of Astragalus Root (5 g), Sinomenium Stem and Rhizome (5 g), Atractylodes Lancea Rhizome (3 g), Jujube (3 g), Glycyrrhiza (1.5 g), and Ginger (1 g). It has been approved by the Japanese Ministry of Health, Labor and Welfare for clinical use in the management of obesity, edema, arthritis, nephritis, and related conditions. In China, BOT is referred to as Fangji Huangqi Tang and has been clinically used for the prevention and treatment of chronic inflammatory diseases [18]^.^ Previous studies have shown that Boiogito is effective in treating chronic inflammation [19,20] and metabolic diseases [21]; however, its mechanism of action on obesity and chronic inflammation remains unclear. In particular, it is currently unclear whether Boiogito differentially regulates adipogenesis and inflammatory responses in different adipocyte differentiation states when treating obesity.

In the present study, we investigated the effects of BOT on adipocyte function and inflammatory responses using both differentiating and mature adipocyte models under TNF-α-induced inflammatory conditions. By incorporating distinct adipocyte developmental stages, we aimed to better reflect the complexity of the adipose tissue microenvironment and to determine whether the regulatory effects of BOT are maintained throughout adipocyte development. Our findings demonstrate that BOT preserves adipogenic capacity, maintains adipocyte function, and attenuates inflammatory responses under inflammatory stress in both differentiating and mature adipocytes. These results highlight the potential role of BOT in maintaining adipose tissue homeostasis and mitigating obesity-associated metabolic inflammation.

## 2. Materials and Methods

### 2.1. Cell Culture and Media

The BOT extract was obtained from Tsumura & Co. (Tokyo, Japan). The HPLC chromatogram of BOT is shown in Supplementary Figure S25. TNF-α and BAY11-7085 were purchased from Fujifilm Wako (Osaka, Japan) and TargetMol (Boston, MA, USA), respectively. 3T3-L1 preadipocytes (ATCC CL-173™, American Type Culture Collection, Waltham, MA, USA) were used to establish an in vitro adipocyte differentiation model. Cells were maintained in Dulbecco’s modified Eagle’s medium (ATCC 30-2002™, Waltham, MA, USA) supplemented with 10% bovine calf serum (BCS; ATCC 30-2030™, Waltham, MA, USA) as basal medium. Cells were cultured at 37 °C in a humidified incubator containing 5% CO_2_.

For routine culture, 3T3-L1 cells were plated in 75 cm^2^ culture flasks at a density of 1 × 10^6^ cells per flask and maintained in 15 mL of basal medium. When the cells reached ~70–80% confluence, they were detached using 0.25% trypsin (Gibco, Thermo Fisher Scientific, Waltham, MA, USA) and subcultured for subsequent experiments. For different assays, cells were seeded as follows: 2 × 10^4^ cells/well in 96 well for Cell Counting Kit-8; 4 × 10^4^ cells/well in 48-well plates for Oil Red O staining and ELISA, 1 × 10^6^ cells/dish for RT-qPCR; and 8 × 10^4^ cells/well in 6-well plates for Western blot analysis. All experiments were performed using at least three independent biological replicates.

### 2.2. Differentiation of 3T3-L1 Cells

Adipogenic differentiation of 3T3-L1 cells was induced according to the ATCC protocol for CL-173™(3T3-L1) and relevant references [22], with minor modifications. To induce adipocyte differentiation, cells were grown to 100% confluence. Differentiation was initiated by replacing the growth medium with differentiation induction medium consisting of DMEM supplemented with 10% fetal bovine serum (ATCC 30-2020, Waltham, MA, USA), 1.0 μM dexamethasone (Fujifilm, Osaka, Japan), 0.5 mM 3-isobutyl-1-methylxanthine (IBMX; Cayman Chemical, Michigan, USA,), and 1.0 μg/mL insulin (Osaka, Japan). After two days of induction, the medium was replaced with adipocyte maintenance medium containing DMEM supplemented with 10% FBS and 1.0 μg/mL insulin (Day 0). The medium was subsequently refreshed every 3 days until recovering mature adipocytes (Day 9).

### 2.3. Experimental Design

We used two drug administration methods.

#### 2.3.1. Differentiation Model

3T3-L1 cells were cultured as described in Section 2.2. On day 0, cells were divided into four groups: control (no TNF-α), TNF-α model (5 ng/mL), and TNF-α plus BOT treatment (5 ng/mL TNF-α in combination with BOT at 0.25 or 0.5 mg/mL). The medium was replaced every 3, days maintaining the respective treatments. On day 9, intracellular lipid accumulation was assessed; MCP-1 and IL-6 levels in culture supernatants were measured by ELISA; mRNA expression of adipogenic markers (PPARγ and C/EBPα) was analyzed by RT-qPCR; and protein expression of NF-κB pathway components (P-p65, p65, IκBα, and P-IκBα) was determined by Western blotting.

#### 2.3.2. Mature Adipocyte Model

3T3-L1 cells were differentiated to maturity (day 9) as described in Section 2.2. Then, cells were switched to serum-free medium and subjected to different treatments. We divided the cells into four groups: control (no TNF-α), TNF-α model (5 ng/mL), and BOT treatment groups (5 ng/mL TNF-α in combination with BOT at 0.25 or 0.5 mg/mL). On day 11, intracellular lipid accumulation was assessed; MCP-1 and IL-6 levels in culture supernatants were measured by ELISA; mRNA expression of adipogenic markers (PPARγ and C/EBPα) was analyzed by RT-qPCR; and protein expression of NF-κB pathway components (P-p65, p65, IκB, and P-IκB) was determined by Western blotting.

### 2.4. Cell Viability Assessment

3T3-L1 cells were seeded in 96-well microplates containing 100 µL of basal medium and cultured at 37 °C and 5% CO_2_ for 2–3 days until 100% confluence. Then, the medium was replaced with a differentiation medium and cultured for 2 days to induce differentiation. Afterward, the cells were cultured in maintenance medium containing the following compounds, used alone or in combination: BOT (0.125–1 mg/mL), TNF-α (5–20 ng/mL), BAY11-7085 (2.5–15 μM), TNF-α (5 ng/mL) + BOT (0.25 and 0.5 mg/mL), BAY (5 μM) + BOT (0.25 and 0.5 mg/mL), and BAY (5 μM) + TNF-α (5 ng/mL). Cells were cultured at 37 °C and 5% CO_2_ for another 9 days, and the medium was changed every three days. On day 9, 10 µL/well of Cell Counting Kit-8 (CCK-8) reagent was added to each well and cells were cultured for 2 h. The absorbance at 450 nm was then measured to determine cell viability.

### 2.5. Oil Red O Staining

Oil Red O staining and extraction-based semiquantification of intracellular lipid accumulation were performed as previously described, with minor modifications [23,24]. Before Oil Red O staining, adipocytes in each group were photographed under a microscope to observe cell morphology. After removing the culture medium, adipocytes were washed twice with phosphate-buffered saline (PBS; 500 μL per well). Then, cells were incubated with 200 μL of 0.36% Oil Red O solution at room temperature for 15 min to stain intracellular lipid droplets. Excess dye was removed by washing the cells three times with distilled water, and stained cells were photographed under a microscope. Subsequently, the retained dye was eluted with 250 μL of isopropanol and the absorbance of the extracted solution was measured at 540 nm. Relative intracellular lipid accumulation was calculated from the background-corrected Oil Red O absorbance values.

### 2.6. Enzyme-Linked Immunosorbent Assay

Culture supernatants from 3T3-L1 adipocytes treated with TNF-α and BOT were collected and centrifuged to remove debris. The levels of adiponectin, IL-6, and MCP-1 were determined using commercial ELISA kits according to manufacturers’ protocols (Proteintech, Rosemont, IL, USA).

### 2.7. RT-qPCR

Total RNA was extracted from adipocytes using the FastGene™ RNA Basic Kit (Nippon Genetics Co., Tokyo, Japan) according to manufacturer’s instructions. The extracted RNA was reverse-transcribed into complementary DNA using the ReverTra Ace^®^ qPCR RT Kit (Toyobo Co., Osaka, Japan). Quantitative real-time PCR was subsequently performed using an Applied Biosystems^®^ 7500 Fast Real-Time PCR System (Thermo Fisher Scientific, Waltham, MA, USA) to determine gene expression levels, which were normalized to β-actin, and relative mRNA expression was calculated using the 2^−^ΔΔCt method.

The primer sequences used for RT-qPCR were adopted from a previously published study [25]. Their in silico specificity was evaluated using NCBI Primer-BLAST against the Mus musculus RefSeq mRNA database, and the sequences are listed in Table 1.

### 2.8. Western Blotting

Protein expression was analyzed using Western blotting. To further validate the involvement of the NF-κB signaling pathway, a specific NF-κB inhibitor BAY11-7085 (5 μM) was used as a positive control. Only a BOT concentration of 0.5 mg/mL was used in the pathway validation experiment, since this dose showed the most significant anti-inflammatory effect in previous experiments.

Total protein was extracted using RIPA lysis buffer containing protease and phosphatase inhibitors, and protein concentration was determined using a BCA assay. Equal amounts of protein were separated by SDS-PAGE and transferred onto PVDF membranes. After blocking, membranes were incubated with primary and secondary antibodies. Protein signals were detected using an imaging system and quantified using ImageJ 1.54g. Antibody information is provided in Table 2.

### 2.9. Statistical Analysis

Statistical analyses were performed using GraphPad Prism 8.0. All experiments were performed at least in triplicate. Data are presented as the mean ± standard deviation. One-way analysis of variance was used to assess differences among multiple groups with a single independent variable, followed by Tukey’s post hoc test for multiple comparisons. A *p* value < 0.05 was considered statistically significant.

## 3. Results

### 3.1. Cytotoxicity During Adipocyte Differentiation

**Figure 1:**
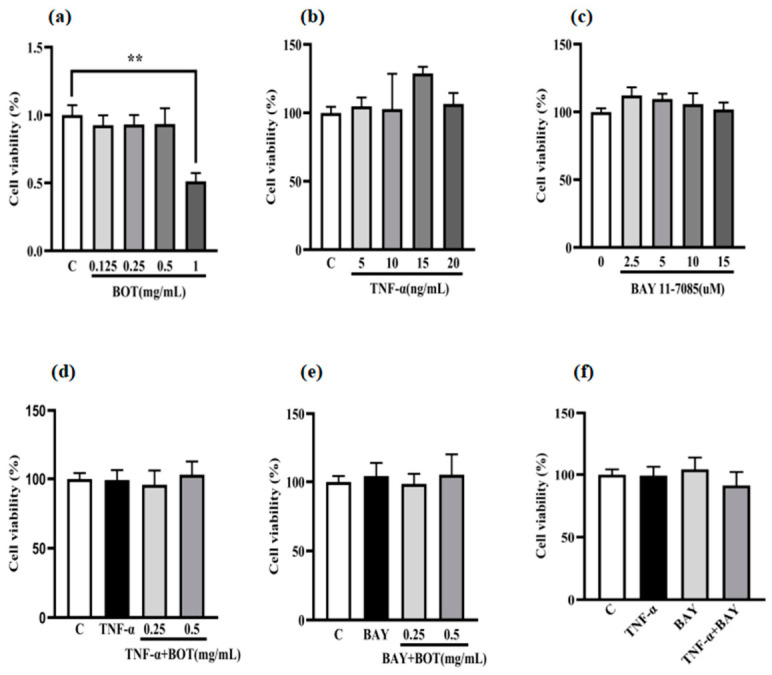
Effects of BOT and combination treatments on cell viability during adipocyte differentiation. (**a**) BOT, (**b**) TNFα, (**c**) BAY11-7085, (**d**) TNF-α (5 ng/mL) + BOT, (**e**) BAY (5 μM) + BOT, and (**f**) TNF-α (5 ng/mL) + BAY (5 μM). Data are presented as mean ± SD (*n* = 5). * *p* < 0.05, ** *p* < 0.01, *** *p* < 0.001 vs. control.

Before examining the effects of BOT on adipocyte differentiation, we first assessed its potential cytotoxicity during the 9-day differentiation period of adipocytes. Cells were treated with the indicated concentration ranges and cell viability was measured (Figure 1). As shown in Figure 1a, BOT exhibited no apparent cytotoxicity at concentrations of 0.125 to 0.5 mg/mL, whereas a significant reduction in cell viability was observed at 1 mg/mL. TNF-α and BAY11-7085 showed no significant cytotoxicity at concentrations of 5–20 ng/mL and 2.5–15 μM, respectively (Figure 1b,c). Treatment with TNFα, BAY11-7085 (BAY), TNFα + BOT, BAY + BOT, or TNFα + BAY had no significant effect on cell viability (Figure 1d–f). These results indicate that the observed cellular responses were attributable to the pharmacological effects of the tested agents rather than nonspecific cytotoxicity. Based on these findings and previous adipocyte studies [26,27], BOT at 0.25 and 0.5 mg/mL, TNF-α at 5 ng/mL, and BAY11-7085 5 μM were selected for the subsequent experiments.

### 3.2. Effects of BOT on Inflammation During Adipocyte Differentiation

#### 3.2.1. BOT Modulates Lipid Accumulation and Adiponectin Secretion

To investigate the effects of BOT under inflammatory conditions, adipocytes were stimulated with TNF-α during differentiation.

**Figure 2:**
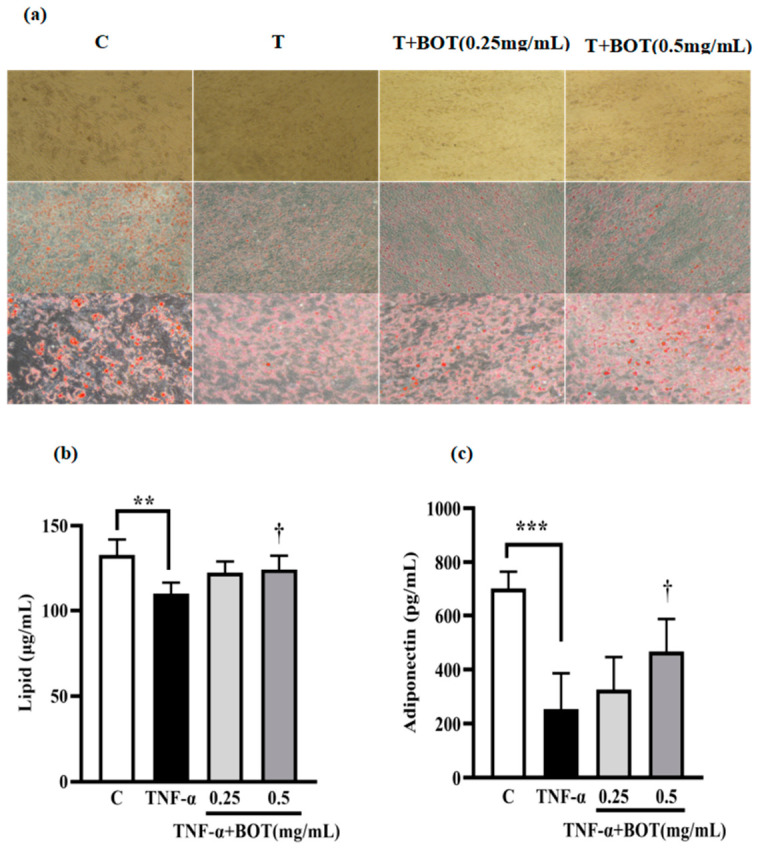
Effects of BOT on lipid accumulation and adiponectin secretion in TNF-α-treated differentiating adipocytes. (**a**) Representative microscopic images of adipocytes from different treatment groups before and after Oil Red O staining. The upper row shows cell morphology before Oil Red O staining. The middle and lower rows show intracellular lipid accumulation after Oil Red O staining at different magnifications. C, control group; T, TNF-α treated group; T + BOT (0.25 mg/mL) and T + BOT (0.5 mg/mL), cells treated with TNF-α in combination with different concentrations of BOT. Red lipid droplets indicate intracellular lipid accumulation. The upper, middle, and lower panels were captured at ×40, ×100, and ×200 magnification, respectively. Scale bar = 300 μm. (**b**) Lipid accumulation. (**c**) Adiponectin secretion. Data are presented as mean ± SD (*n* = 6). * *p* < 0.05, ** *p* < 0.01, *** *p* < 0.001 vs. control; † *p* < 0.05, †† *p* < 0.01, ††† *p* < 0.001 vs. TNF-α.

As shown in Figure 2a, differences in intracellular lipid accumulation were observed among the groups, particularly after Oil Red O staining. The control group displayed marked accumulation of red-stained lipid droplets, whereas the TNF-α group showed fewer lipid droplets and weaker staining intensity. Compared with the TNF-α group, cells treated with BOT under TNF-α-treated conditions exhibited increased red-stained lipid droplets, especially at 0.5 mg/mL.

Furthermore, quantitative analysis of Oil Red staining and adiponectin secretion showed the same trend. Compared with the control group, TNF-α suppressed lipid accumulation (109.8 ± 6.57 vs. 132.9 ± 9.04 μg/mL, *p* <0.01) and reduced adiponectin secretion (253.6 ± 132.3 vs.700.6 ± 61.79 pg/mL, *p* < 0.001) (Figure 2b,c). Notably, BOT treatment significantly attenuated the inhibitory effects induced by TNF-α, resulting in a dose-dependent restoration of lipid accumulation (*p* < 0.05 vs. TNF-α group). Meanwhile, increasing BOT concentrations progressively increased adiponectin secretion (*p* < 0.05 vs. TNF-α group). These findings indicate that BOT may modulate lipid accumulation and adiponectin secretion in adipocytes under TNF-α-treated conditions.

#### 3.2.2. BOT Regulates Adipogenic Gene Expression

To explore whether the effects of BOT on lipid accumulation and adiponectin secretion are mediated at the transcriptional level, the expression of adipogenesis-related genes was examined.

**Figure 3:**
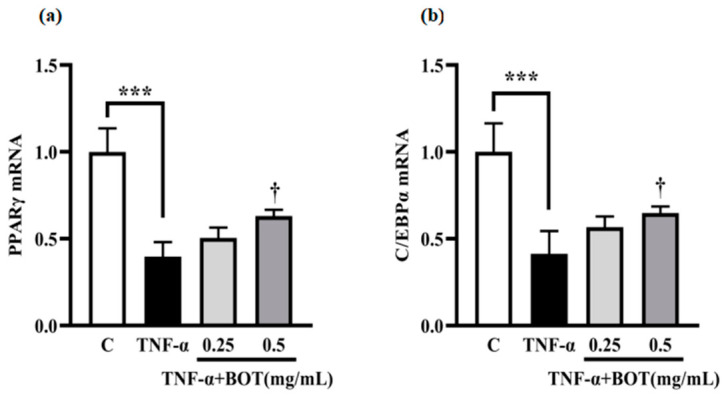
Effects of BOT on adipogenic gene expression in TNF-α-treated differentiating adipocytes. (**a**) PPARγ mRNA expression. (**b**) C/EBPα mRNA expression. Data are presented as mean ± SD (*n* = 4). * *p* < 0.05, ** *p* < 0.01, *** *p* < 0.001 vs. control;† *p* < 0.05, †† *p* < 0.01, ††† *p* < 0.001 vs. TNF-α.

As shown in Figure 3, under inflammatory conditions, TNF-α stimulation suppressed PPARγ mRNA and C/EBPα mRNA expression to 0.40-fold and 0.41-fold of the control, respectively (both *p* < 0.001). Notably, BOT treatment partially reversed this suppression in a dose-dependent manner. At 0.5 mg/mL, BOT increased PPARγ and C/EBPα expression by 0.58-fold and 0.61-fold, respectively, compared with the TNF-α group (*p* < 0.05; Figure 3a,b).

#### 3.2.3. BOT Suppresses Inflammatory Cytokine Production

To evaluate the anti-inflammatory effects of BOT, MCP-1 and IL-6 levels in the culture medium were quantified using ELISA.

**Figure 4:**
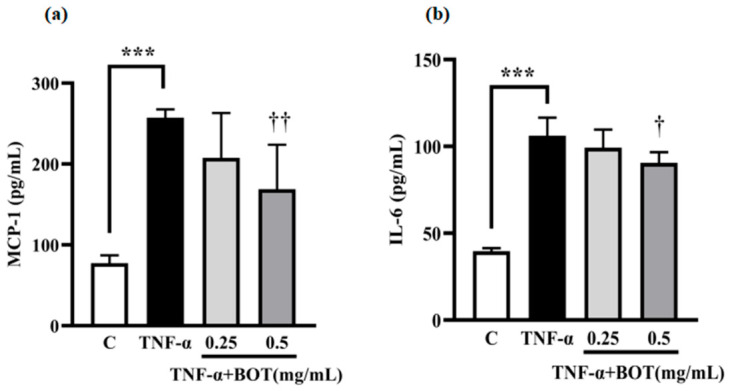
Effects of BOT on inflammatory cytokine production in TNF-α-treated differentiating adipocytes. (**a**) MCP-1 concentration. (**b**) IL-6 levels concentration. Data are presented as mean ± SD (MCP-1: *n* = 6; IL-6: *n* = 5). * *p* < 0.05, ** *p* < 0.01, *** *p* < 0.001 vs. control; † *p* < 0.05, †† *p* < 0.01, ††† *p* < 0.001 vs. TNF-α.

Upon TNF-α stimulation, MCP-1 and IL-6 levels increased compared with the untreated control group (MCP-1: 257.4 ± 10.21 vs. 77.30 ± 9.89 pg/mL, *p* < 0.01; IL-6: 106.2 ± 10.37 vs. 39.69 ± 1.77 pg/mL, *p* < 0.01; Figure 4a,b).

Importantly, BOT exhibited a dose-dependent inhibitory effect under TNF-α-induced inflammation. With increasing BOT concentrations, MCP-1 and IL-6 levels progressively decreased (MCP-1: 207.6 ± 55.37 and 168.9 ± 54.86 pg/mL; IL-6: 99.35 ± 10.37 and 90.59 ± 6.04 pg/mL), being significantly lower than those in the TNF-α-treated group (*p* < 0.05). At the highest concentration, BOT reduced MCP-1 and IL-6 levels by ~34.4% and 14.7%, respectively.

#### 3.2.4. BOT Inhibits NF-κB Signaling

To investigate the molecular mechanisms underlying the anti-inflammatory effects of BOT, NF-κB signaling was assessed using Western blotting. The original uncropped Western blot images are shown in Supplementary Figures S1–S24.

**Figure 5:**
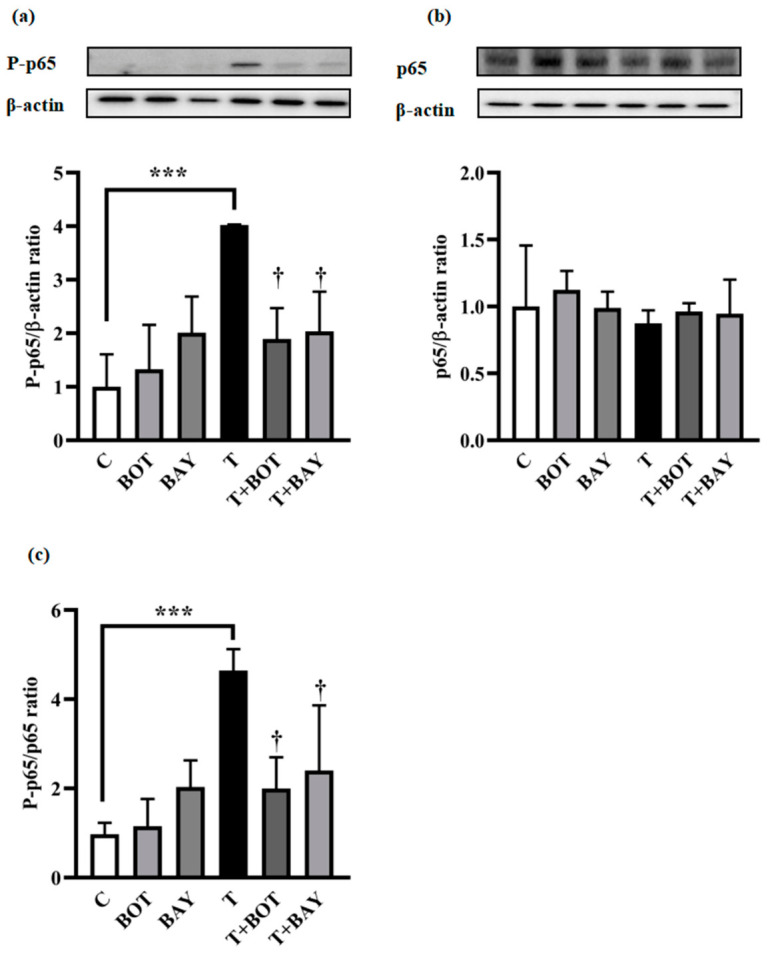

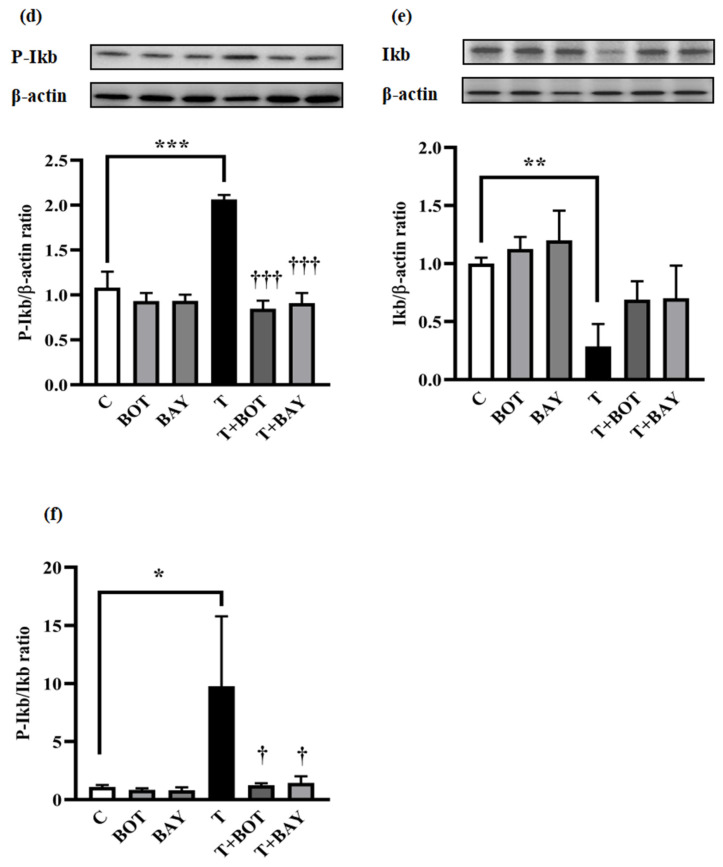
Effects of BOT and BAY11-7085 on TNF-α-induced NF-κB signaling activity during adipocyte differentiation. Western blot lanes from left to right: C, untreated control group; BOT, cells treated with BOT (0.5 mg/mL); BAY, cells treated with the NF-κB inhibitor BAY11-7085 (5 μM); T, cells stimulated with TNF-α (5 ng/mL); T + BOT, cells co-treated with TNF-α (5 ng/mL) and BOT (0.5 mg/mL); and T + BAY, cells co-treated with TNF-α (5 ng/mL) and BAY11-7085 (5 μM). (**a**) Phosphorylated p65 (P-p65) expression. (**b**) Total p65 expression. (**c**) P-p65/p65 ratio. (**d**) Phosphorylated IκB (P-IκB). (**e**) Total IκB expression. (**f**) P-IκB/IκB ratio. Data are presented as mean ± SD (n = 3). * *p* < 0.05, ** *p* < 0.01, *** *p* < 0.001 vs. control; † *p* < 0.05, †† *p* < 0.01, ††† *p* < 0.001 vs. TNF-α.

Compared with the control group, TNF-α stimulation significantly increased the phosphorylation levels of p65, whereas co-treatment with BOT or BAY11-7085 noticeably attenuated TNFα-induced P-p65 expression (*p* < 0.05; Figure 5a). However, no significant differences in total NF-κB p65 protein expression were observed among the treatment groups, indicating that BOT, BAY11-7085, and TNFα treatments did not affect total NF-κB protein levels (*p* > 0.05; Figure 5b). Furthermore, analysis of the P-p65/p65 ratio confirmed that BOT effectively inhibited TNFα-induced NF-κB activation, with an inhibitory effect comparable to that of BAY11-7085 (*p* < 0.05; Figure 5c).

As shown in Figure 5d, TNFα treatment significantly increased P-IκB protein levels, whereas treatment with BOT or BAY11-7085 alone had no significant effect on P-IκB expression. However, co-treatment with BOT or BAY11-7085 markedly suppressed the TNFα-induced increase in P-IκB levels (*p* < 0.001). In addition, analysis of IκBα protein expression revealed that TNFα significantly reduced IκB protein levels compared with the control group, while co-treatment with BOT or BAY11-7085 significantly reversed the TNFα-induced reduction in IκB expression (*p* < 0.05; Figure 5e). Furthermore, analysis of the P-IκB/IκB ratio confirmed that BOT effectively inhibited TNFα-induced IκB phosphorylation, with an effect comparable to that of the NF-κB inhibitor BAY11-7085 (*p* < 0.05; Figure 5f).

### 3.3. Effects of BOT on Inflammation in Mature Adipocytes

To investigate whether BOT differentially regulates adipogenesis and inflammatory responses under different adipocyte differentiation states, we conducted the same experiment using mature adipocytes.

#### 3.3.1. BOT Modulates Lipid Accumulation and Adiponectin Secretion

**Figure 6:**
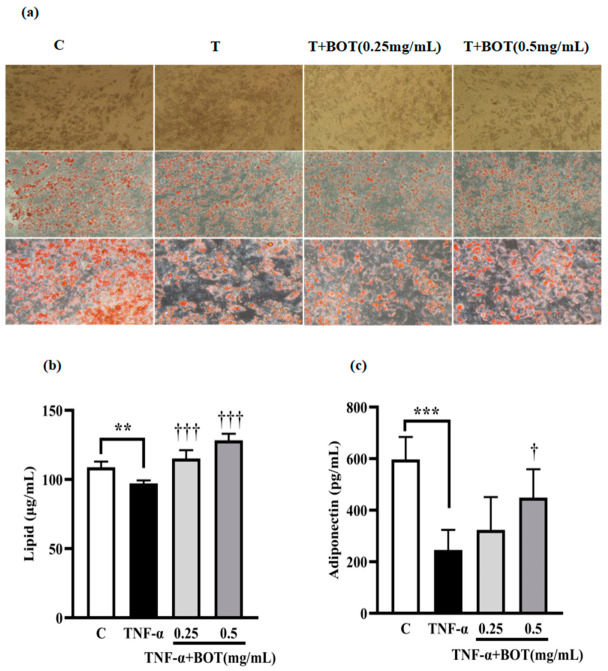
Effects of BOT on lipid accumulation and adiponectin secretion in TNF-α-treated mature adipocytes. (**a**) Representative microscopic images of adipocytes from different treatment groups before and after Oil Red O staining. The upper row shows cell morphology before Oil Red O staining. The middle and lower rows show intracellular lipid accumulation after Oil Red O staining at different magnifications. C, control group; T, TNF-α-treated group; T + BOT (0.25 mg/mL) and T + BOT (0.5 mg/mL), cells treated with TNF-α in combination with different concentrations of BOT. Red lipid droplets indicate intracellular lipid accumulation. The upper, middle, and lower panels were captured at ×40, ×100, and ×200 magnification, respectively. Scale bar = 300 μm. (**b**) Lipid accumulation. (**c**) Adiponectin secretion. Data are presented as mean ± SD (*n* = 6). * *p* < 0.05, ** *p* < 0.01, *** *p* < 0.001 vs. control; † *p* < 0.05, †† *p* < 0.01, ††† *p* < 0.001 vs. TNF-α.

As shown in Figure 6a, differences in intracellular lipid accumulation were observed among the groups, particularly after Oil Red O staining. The control group displayed marked accumulation of red-stained lipid droplets, whereas the TNF-α group showed fewer lipid droplets and weaker staining intensity. Compared with the TNF-α group, cells treated with BOT under TNF-α-treated conditions exhibited increased red-stained lipid droplets, especially at 0.5 mg/mL.

Furthermore, quantitative analysis of Oil Red staining and adiponectin secretion showed the same trend. Compared with the control group, TNF-α significantly suppressed lipid accumulation (97.17 ± 2.17 vs. 108.8 ± 4.22 μg/mL, *p* < 0.01) and adiponectin secretion (245.9 ± 77.56 vs. 597.3 ± 87.24 pg/mL, *p* < 0.001). BOT dose-dependently restored these parameters, with lipid levels increasing to 115.2 ± 5.94 and 128.4 ± 4.71 μg/mL and adiponectin levels to 323.9 ± 127.4 and 449.0 ± 110.0 pg/mL at 0.25 and 0.5 mg/mL, respectively (Figure 6b,c).

#### 3.3.2. BOT Regulates Adipogenic Gene Expression

**Figure 7:**
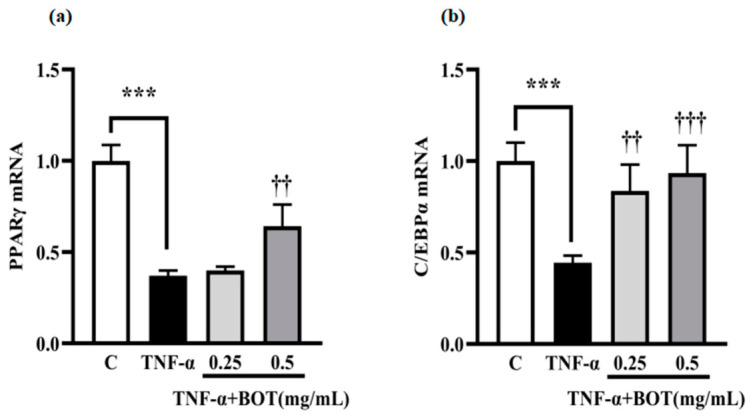
Effects of BOT on adipogenic gene expression in TNF-α-treated mature adipocytes. (**a**) PPARγ mRNA expression. (**b**) C/EBPα mRNA expression. Data are presented as mean ± SD (*n* = 4). * *p* < 0.05, ** *p* < 0.01, *** *p* < 0.001 vs. control; † *p* < 0.05, †† *p* < 0.01, ††† *p* < 0.001 vs. TNF-α.

At the transcriptional level, TNF-α downregulated the mRNA expression of PPARγ and C/EBPα compared with the control group (~60% reduction, *p* < 0.001). BOT significantly reversed these effects, particularly at 0.5 mg/mL, where PPARγ mRNA and C/EBPα mRNA expression levels were restored to ~70% and 1-fold relative to the TNF-α group, respectively (Figure 7a,b).

#### 3.3.3. BOT Suppresses Inflammatory Cytokine Production

**Figure 8:**
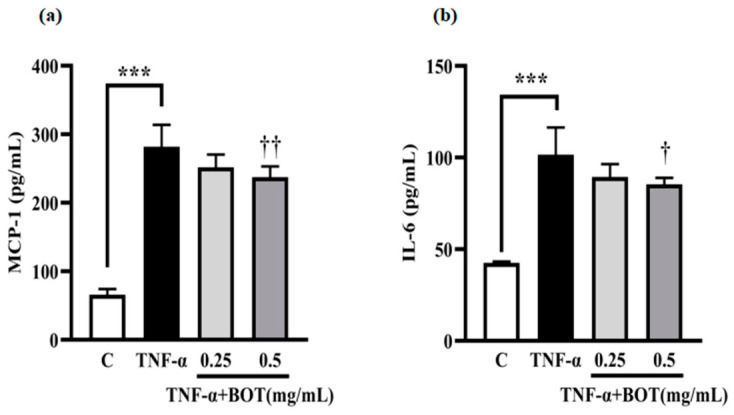
Effects of BOT on inflammatory cytokine production in TNF-α-treated mature adipocytes. (**a**) MCP-1 concentration. (**b**) IL-6 concentration. Data are presented as mean ± SD (MCP-1: *n* = 6; IL-6: *n* = 5). * *p* < 0.05, ** *p* < 0.01, *** *p* < 0.001 vs. control; † *p* < 0.05, †† *p* < 0.01, ††† *p* < 0.001 vs. TNF-α.

Furthermore, Figure 8 shows the experimental results. At the level of inflammatory cytokines, BOT dose-dependently suppressed TNF-α-induced production of inflammatory cytokines, including MCP-1 (251.9 ± 18.66 and 237.6 ± 15.65 pg/mL at 0.25 and 0.5 mg/mL, respectively; *p* < 0.01) and IL-6 (89.44 ± 6.98 and 85.45 ± 3.52 pg/mL, respectively; *p* < 0.05).

#### 3.3.4. BOT Inhibits NF-κB Signaling

**Figure 9:**
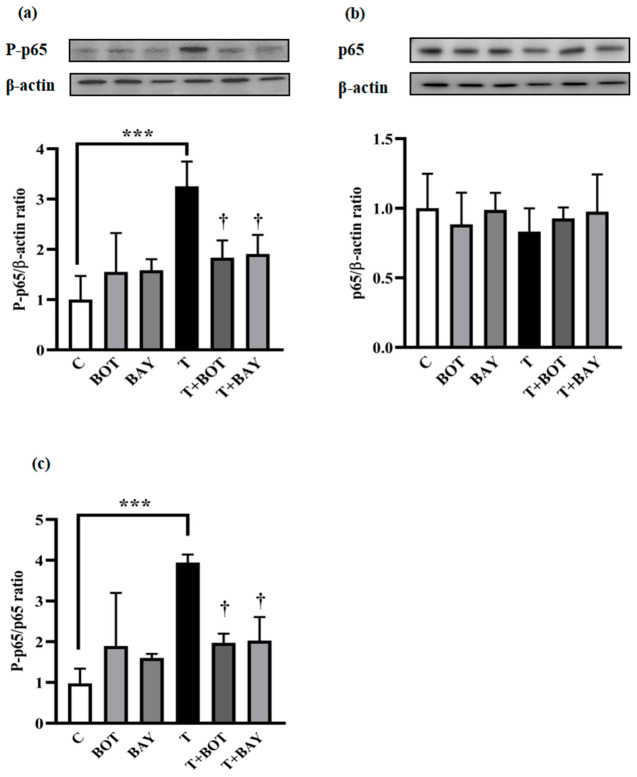

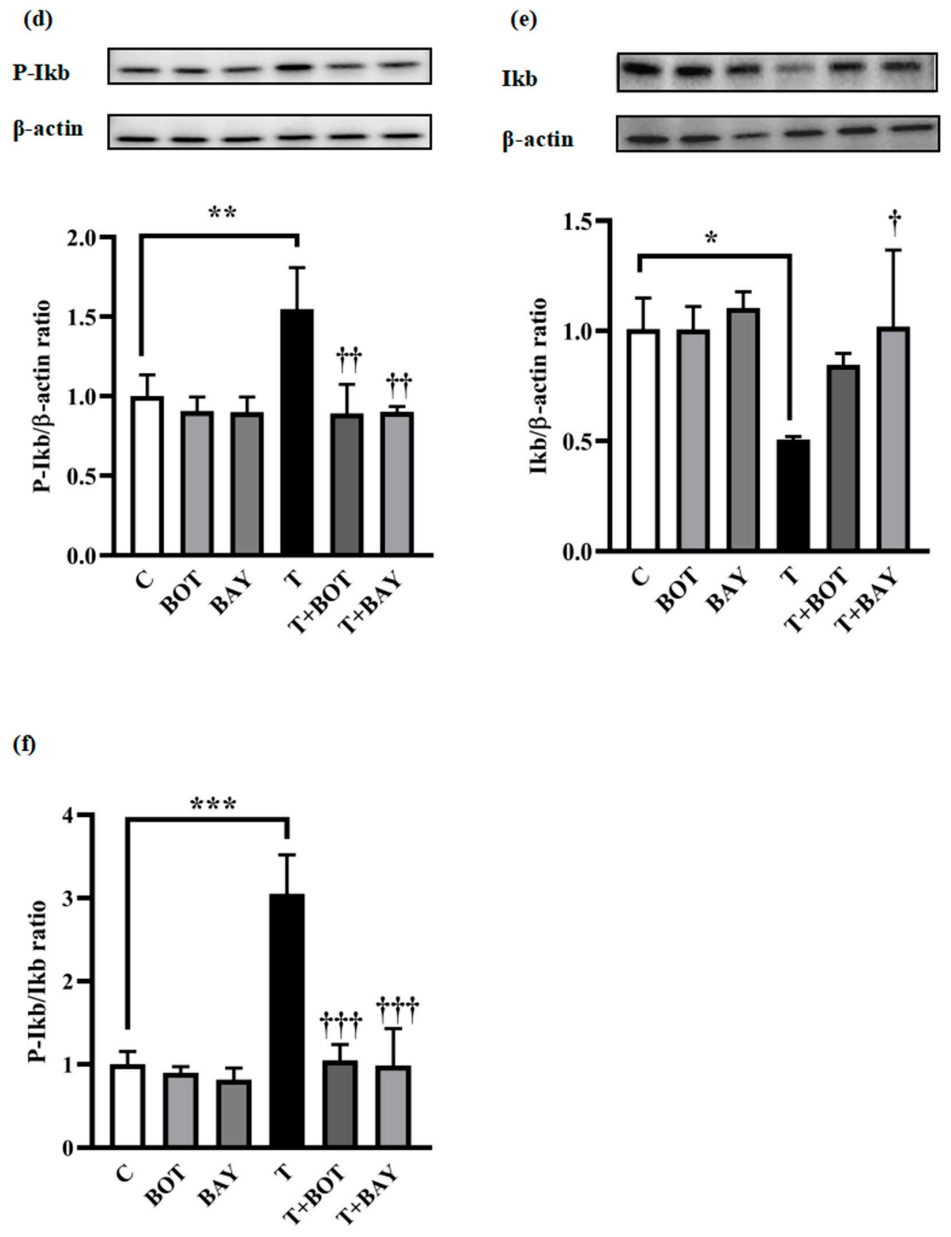
Effects of BOT and BAY11-7085 on TNF-α-induced NF-κB signaling activity in mature adipocytes. Western blot lanes from left to right: C, untreated control group; BOT, cells treated with BOT (0.5 mg/mL); BAY, cells treated with the NF-κB inhibitor BAY11-7085 (5 μM); T, cells stimulated with TNF-α (5 ng/mL); T + BOT, cells co-treated with TNF-α (5 ng/mL) and BOT (0.5 mg/mL); and T + BAY, cells co-treated with TNF-α (5 ng/mL) and BAY11-7085 (5 μM). (**a**) Phosphorylated p65 (P-p65) expression. (**b**) Total p65 expression. (**c**) P-p65/p65 ratio. (**d**) Phosphorylated IκB (P-IκB). (**e**) Total IκB expression. (**f**) P-IκB/IκB ratio. Data are presented as mean ± SD (n = 3). * *p* < 0.05, ** *p* < 0.01, *** *p* < 0.001 vs. control; † *p* < 0.05, †† *p* < 0.01, ††† *p* < 0.001 vs. TNF-α.

Western blot analysis results are shown in Figure 9. TNFα markedly induced p65 phosphorylation, whereas co-treatment with BOT or BAY11-7085 noticeably attenuated TNFα-induced P-p65 expression (*p* < 0.05; Figure 9a). However, no significant differences in total NF-κB p65 protein expression were observed among the treatment groups, indicating that BOT, BAY11-7085, and TNFα treatments did not affect total NF-κB protein levels (*p* > 0.05; Figure 9b). Furthermore, analysis of the P-p65/p65 ratio confirmed that BOT effectively inhibited TNFα-induced NF-κB activation, with an inhibitory effect comparable to that of BAY11-7085 (*p* < 0.05; Figure 9c).

As shown in Figure 9d, TNFα treatment significantly increased P-IκB protein levels, whereas treatment with BOT or BAY11-7085 alone had no significant effect on P-IκB expression. However, co-treatment with BOT or BAY11-7085 markedly suppressed the TNFα-induced increase in P-IκB levels (*p* < 0.01). In addition, analysis of IκBα protein expression revealed that TNFα significantly reduced IκB protein levels compared with the control group, while co-treatment with BOT or BAY11-7085 significantly reversed the TNFα-induced reduction in IκB expression (*p* < 0.05; Figure 9e). Furthermore, analysis of the P-IκB/IκB ratio confirmed that BOT effectively inhibited TNFα-induced IκB phosphorylation, with an effect comparable to that of the NF-κB inhibitor BAY11-7085 (*p* < 0.001; Figure 9f).

## 4. Discussion

Our study demonstrates that Boiogito exerts protective effects against TNF-α-induced adipocyte dysfunction in both differentiating and mature adipocytes. Importantly, BOT not only attenuated inflammatory responses but also restored lipid accumulation, adipogenic gene expression, and adiponectin production under inflammatory conditions. These findings suggest that BOT contributes to the maintenance of adipocyte homeostasis throughout different stages of adipocyte development. Given the central role of adipocyte dysfunction in obesity-associated metabolic inflammation, the ability of BOT to preserve adipocyte function may represent an important mechanism underlying its beneficial metabolic effects.

During adipocyte differentiation, TNF-α markedly suppressed lipid accumulation and reduced the expression of the key adipogenic transcription factors PPARγ and C/EBPα. These findings are consistent with recent studies demonstrating that TNF-α impairs adipogenesis through activation of inflammatory signaling pathways, particularly NF-κB, thereby inhibiting the transcriptional program required for adipocyte maturation [28,29]. PPARγ and C/EBPα are widely recognized as central regulators of terminal adipocyte differentiation and function, acting cooperatively to maintain adipocyte phenotype and lipid storage capacity [30]. Suppression of these transcription factors disrupts lipid droplet formation and lipid homeostasis, ultimately contributing to adipose tissue dysfunction [31]. In the present study, Boiogito restored the TNF-α-induced reduction in lipid accumulation and upregulated the expression of PPARγ and C/EBPα, suggesting that BOT may improve adipocyte differentiation by preserving the adipogenic program.

In mature adipocytes, TNF-α stimulation likewise reduced the expression of the key adipogenic transcription factors PPARγ and C/EBPα, as well as adiponectin. Because TNF-α acts on already differentiated and functionally stable mature adipocytes, its primary effect is not the inhibition of adipocyte formation, but rather the induction of lipid metabolic dysregulation and chronic inflammatory responses [32,33]. Previous studies have shown that sustained activation of inflammatory signaling pathways by TNF-α disrupts metabolic homeostasis in mature adipocytes and exacerbates inflammatory amplification [34]. In contrast, Boiogito upregulated the expression of PPARγ and C/EBPα, suggesting that BOT may ameliorate TNF-α-induced dysfunction in mature adipocytes by restoring lipid metabolic homeostasis and suppressing inflammatory cascade responses. In addition, the recovery of adiponectin expression further supports the possibility that BOT contributes to the restoration of adipocyte endocrine and metabolic function. Taken together, these findings indicate that BOT exerts protective effects not only on adipocyte differentiation but also on the maintenance of mature adipocyte function, supporting its role in preserving adipose tissue metabolic homeostasis under inflammatory stress.

Notably, TNF-α markedly induced the expression of IL-6 and MCP-1, accompanied by activation of the NF-κB signaling pathway. IL-6 is a key mediator of obesity-associated chronic inflammation, whereas MCP-1 plays a critical role in macrophage recruitment and amplification of adipose tissue inflammation [35,36]. Previous studies have demonstrated that sustained elevation of MCP-1 promotes the infiltration of pro-inflammatory M1 macrophages into adipose tissue, thereby establishing a vicious cycle characterized by continuous production of inflammatory cytokines such as TNF-α and IL-6 [37,38]. In the present study, Boiogito significantly suppressed the expression of IL-6 and MCP-1 while also reducing NF-κB activity. These effects were comparable to those observed with the NF-κB signaling inhibitor BAY11-7085, suggesting that BOT may ameliorate the adipose tissue microenvironment by suppressing NF-κB-mediated chemokine production and subsequent macrophage infiltration.

A representative three-dimensional HPLC fingerprint supplied by the manufacturer, Tsumura & Co., identified several classes of phytochemicals in BOT, including alkaloids such as sinomenine and magnoflorine; flavonoid- and isoflavonoid-related compounds such as liquiritin, liquiritigenin, isoliquiritin, isoliquiritigenin, and formononetin; the triterpenoid saponin glycyrrhizin; and ginger-derived phenolic compounds such as 6-gingerol and 6-shogaol. Previous studies have reported that sinomenine modulates IκB phosphorylation and the nuclear accumulation of NF-κB p65, whereas formononetin attenuates cytokine-induced NF-κB activation [39,40]. In addition, isoliquiritigenin has been shown to suppress inflammatory responses in an adipocyte–macrophage co-culture model and to attenuate adipose tissue inflammation and fibrosis [41]. However, these reports provide only indirect evidence for plausible candidate constituents and do not establish that any individual compound is responsible for the effects of the complete BOT formulation observed in the present study.

Furthermore, TNF-α-induced activation of NF-κB not only promotes the expression of inflammatory cytokines, but also induces abnormal lipolysis and excessive release of free fatty acids (FFA) [42,43]. FFAs are considered important mediators linking obesity, chronic inflammation, and insulin resistance. Excessive FFA and TNF-α have been shown to inhibit GLUT4 expression and induce insulin resistance. As an insulin-dependent glucose transporter, decreased GLUT4 expression leads to impaired glucose uptake, thereby promoting insulin resistance and the development of type 2 diabetes [44]. Therefore, the improvement of inflammatory responses and lipid metabolism abnormalities by BOT suggests that it may further improve insulin sensitivity and has potential therapeutic value for obesity-related metabolic diseases such as insulin resistance and type 2 diabetes mellitus.

Several limitations of this study should be acknowledged. First, our findings indicate that BOT suppresses TNF-α-induced inflammatory responses, although we cannot yet determine whether BOT protects adipocytes against other inflammatory stimuli. Second, the present findings were obtained using in vitro adipocyte models and did not assess metabolic relevant markers; therefore, they may not fully reflect the complex metabolic and immune environment of adipose tissue in vivo. Further animal studies are needed to confirm the protective effects of BOT on adipose tissue dysfunction and metabolic inflammation. Then, fibrosis-related markers, including collagen and fibronectin, and adipocyte size distribution were not evaluated; therefore, whether BOT directly affects adipose tissue fibrosis, remodeling, or adipocyte hypertrophy remains unclear. In addition, although NF-κB signaling appears to contribute to the observed effects, the precise molecular targets and additional regulatory pathways involved in BOT-mediated protection remain to be clarified, such as MAPK and AP-1 pathways. Furthermore, the BOT concentration used in the present experiments may not correspond to a clinically achievable level of systemic exposure. Human pharmacokinetic and pharmacodynamic studies quantifying representative bioactive constituents of BOT and their metabolites are needed to determine the clinical relevance of the effective concentration observed in this study.

## Figures and Tables

**Table 1:** Primer sequences used for RT-qPCR.

Gene	Forward (5′-3′)	Reverse (5′-3′)
PPARγ	TCACAATGCCATCAGGT	GCGGGAAGGACTTTATGTA
C/EBPα	GCCCCTCAGTCCCTGTCTTTA	AGCCCTCCACCTCCCTGTAG
β-actin	CCTCTATGCCAACACAGT	AGCCACCAATCCACACAG

PPARγ, peroxisome proliferator-activated receptor gamma; C/EBPα, CCAAT/enhancer-binding protein alpha. β-actin was used as the internal control gene.

**Table 2:** Antibodies used in this study.

Target Protein	Antibody (Catalog No.)	Dilution	Source
Phospho-NF-κB p65	P-p65 (#3033)	1:1000	Cell Signaling Technology
p65 (total)	p65 (10475-1-AP)	1:1000	Proteintech
Phospho-IκBα	P-IκBα (#2859)	1:1000	Cell Signaling Technology
IκB (total)	IκB (10268-1-AP)	1:1000	Proteintech
β-actin	β-actin (#5125)	1:1000	Cell Signaling Technology
Rabbit IgG (Secondary Antibody)	Anti-rabbit IgG (#7074)	1:2000	Cell Signaling Technology

P-p65 and P-IκBα represent the phosphorylated forms of NF-κB p65 and IκBα, respectively. β-actin was used as an internal loading control. Anti-rabbit IgG was used as the horseradish peroxidase (HRP)-conjugated secondary antibody.

## Data Availability

The data presented in this study are available from the corresponding author upon reasonable request.

## Supplementary Materials

The following supporting information can be downloaded at: https://www.mdpi.com/article/10.3390/cimb48070693/s1.

## Abbreviations

The following abbreviations are used in this manuscript:

BOT	Boiogito
C/EBPα	CCAAT/enhancer-binding protein α
FFA	free fatty acids
IκB	Inhibitor of κB
IL-6	Interleukin-6
MCP-1	Monocyte chemoattractant protein-1
NF-κB	Nuclear factor kappa B
PPARγ	Peroxisome proliferator-activated receptor γ
P-IκB	Phosphorylated IκB
P-p65	Phosphorylated p65
TNF-α	Tumor necrosis factor-α
