# Supplementary material for: Boiogito Ameliorates Inflammation-Associated Adipocyte Dysfunction and Restores Adipogenesis in Association with Suppression of NF-κB Signaling
Source: Curr Issues Mol Biol. 2026 Jul 8;48(7):693. doi: 10.3390/cimb48070693 (PMC13406834; doi:10.3390/cimb48070693)
Supplement: Supplementary file 1 [file cimb-48-00693-s001.zip › cimb-4394620-supplementary.pdf]

Manuscript title: Boiogito Ameliorates Inflammation-Associated Adipocyte Dysfunction and Restores Adipogenesis in Association with Suppression of NF-κB Signaling

Authors: Yi Luo, Ailing Hu, Jingya Lu, Wenshu Yuan, Yu Tan, Takuji Yamaguchi, Zenji Kawakami, Yasushi Ikarashi, Yoshinao Harada, and Hiroyuki Kobayashi

Table of Contents

Figure S1-S24. Original uncropped Western blot images from three independent experiments
Figure S25. Three-dimensional HPLC chromatogram of the BOT extract.

Supplementary Figure S1. p-p65(Replicate1)

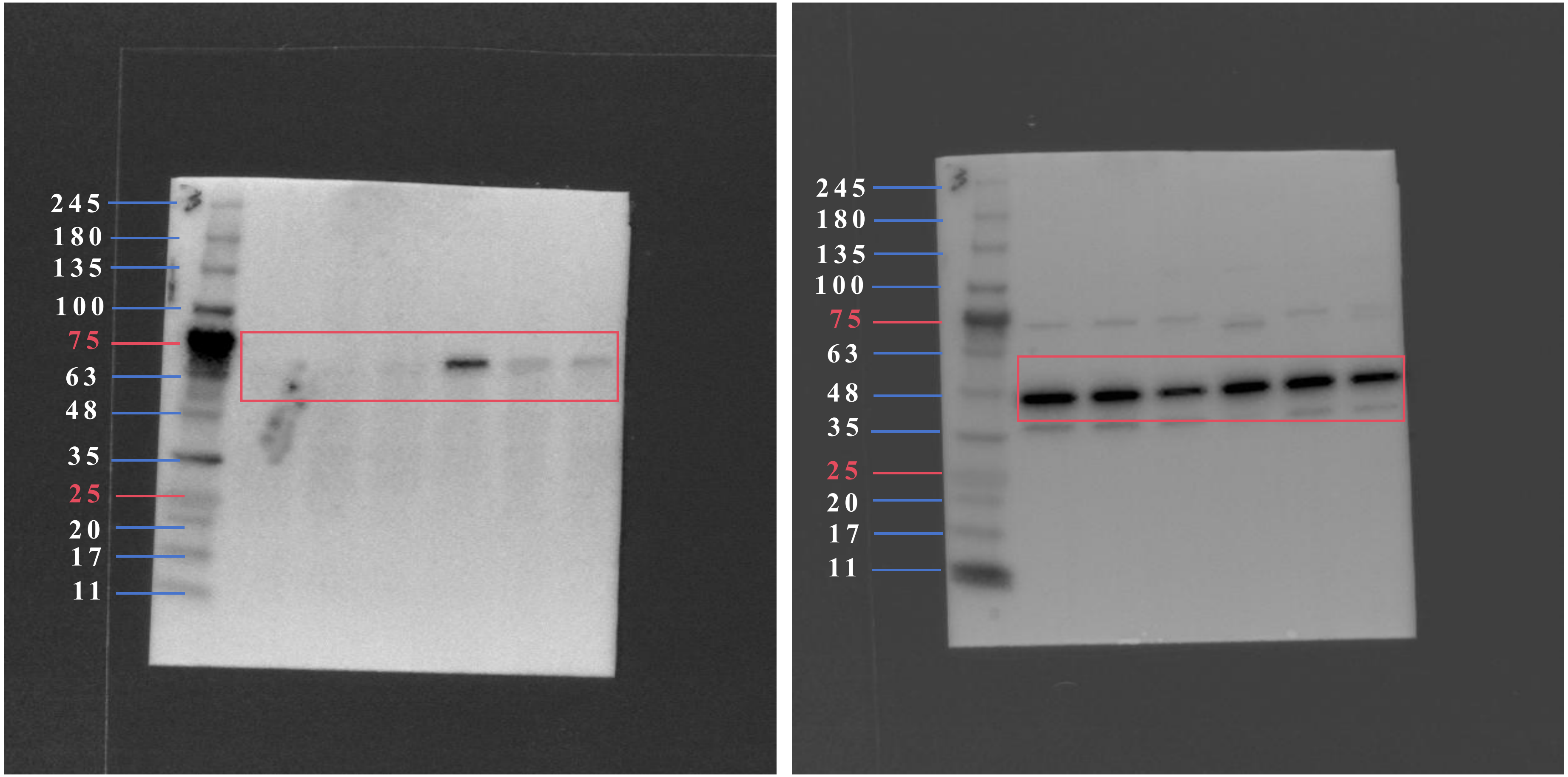

Lane1: Control

Lane2: BOT

Lane3: BAY11-7085

Lane4: TNF-α

Lane5: TNF-α+BOT

Lane6: TNF-α+BAY11-7085

Supplementary Figure S1.
Uncropped Western blot corresponding to Figure 5 (a), (p-p65), Replicate 1.Target protein and β-actin were detected on the same membrane using the same protein lysates. The images show the full membranes used to generate the main figure. Red boxes indicate the cropped regions presented in the manuscript. All lanes are shown. No brightness or contrast adjustments were applied to individual bands.

Supplementary Figure S2. p-p65(Replicate2)

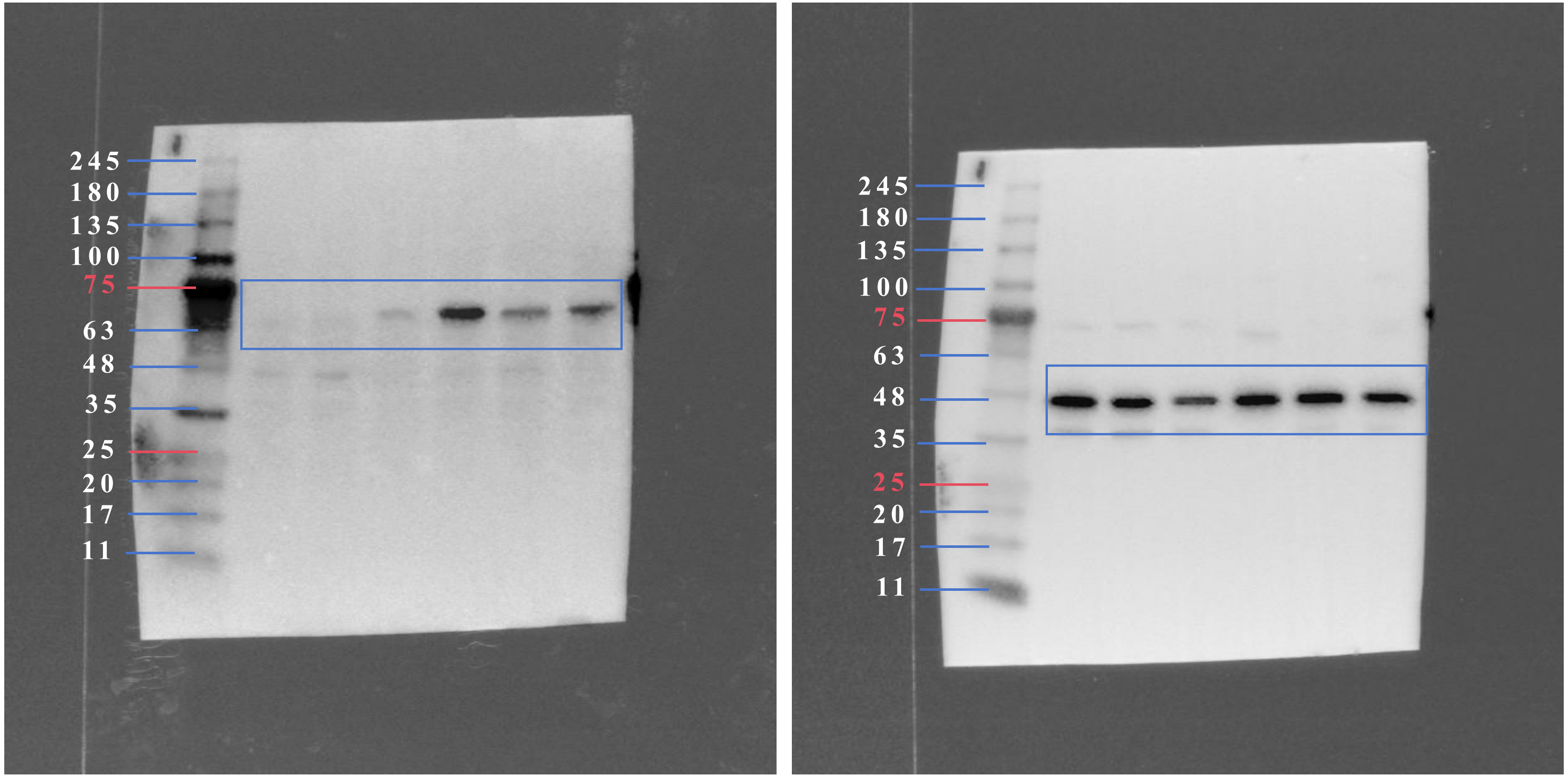

Lane1: Control

Lane2: BOT

Lane3: BAY11-7085

Lane4: TNF-α

Lane5: TNF-α+BOT

Lane6: TNF-α+BAY11-7085

Supplementary Figure S2.
Uncropped Western blot corresponding to Figure 5 (a) (p-p65), Replicate 2. Target protein and β-actin were detected on the same membrane using the same protein lysates. The images show the full membranes from an independent biological replicate used for quantitative densitometric analysis presented in Figure 5 (a). All lanes are shown. No brightness or contrast adjustments were applied to individual bands.

Supplementary Figure S3. p-p65(Replicate3)

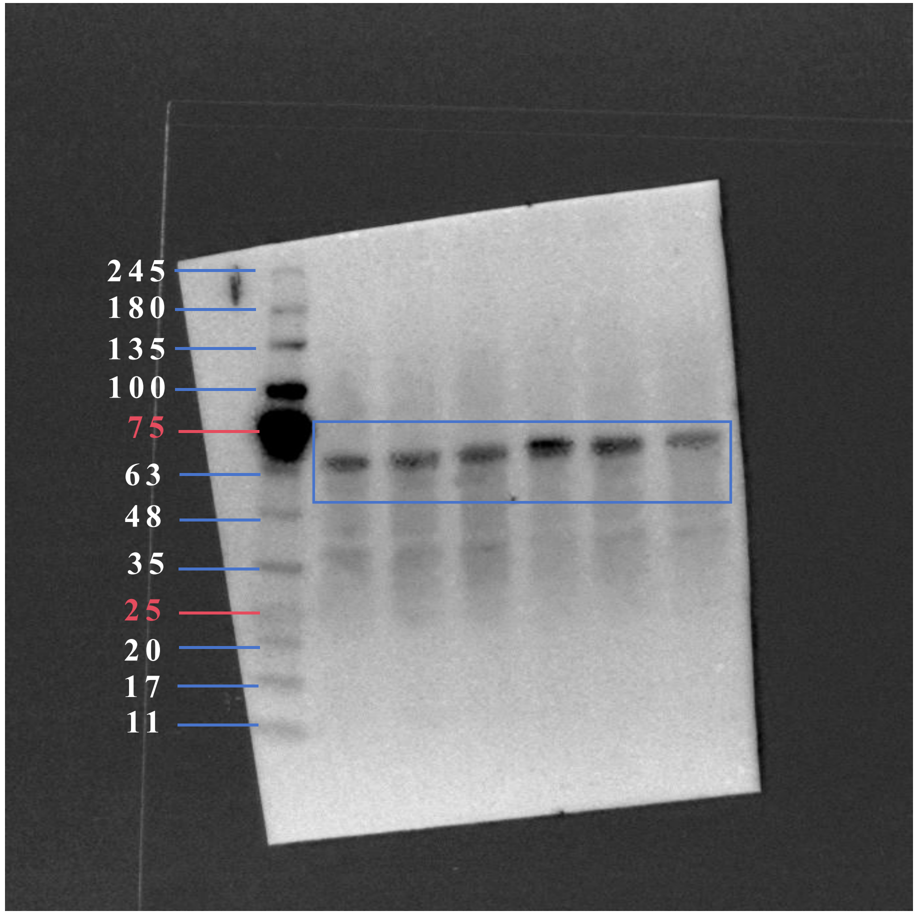

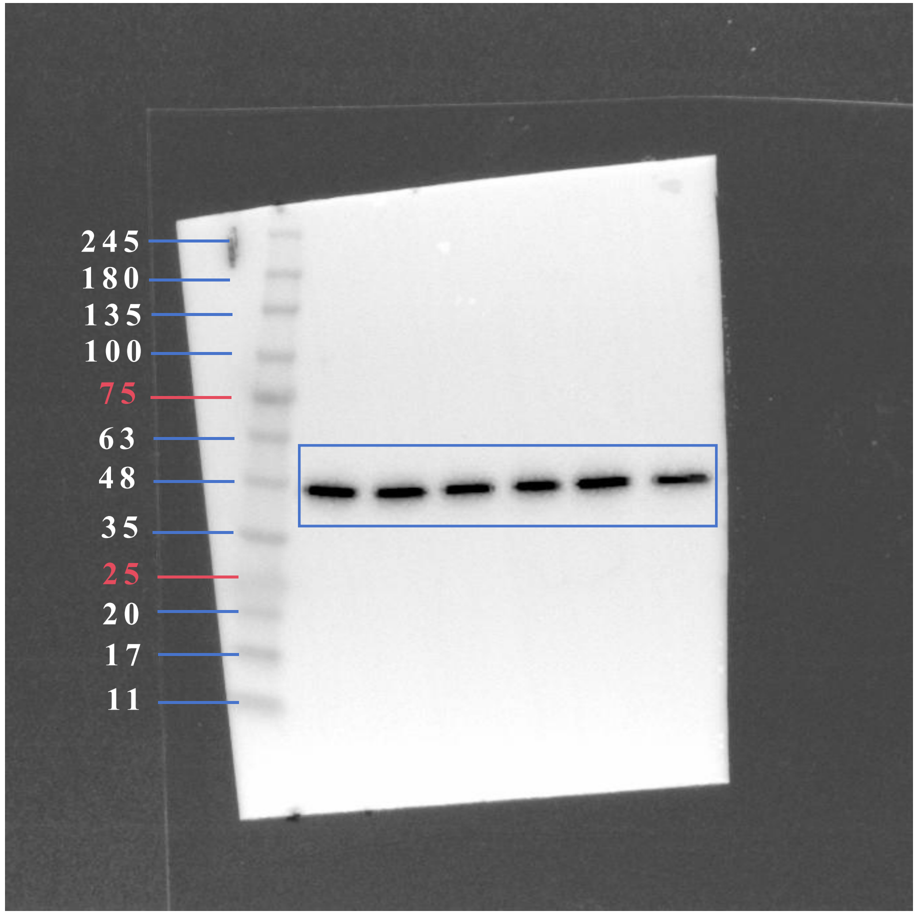

Lane1: Control

Lane2: BOT

Lane3: BAY11-7085

Lane4: TNF-α

Lane5: TNF-α+BOT

Lane6: TNF-α+BAY11-7085

Supplementary Figure S3.
Uncropped Western blot corresponding to Figure 5 (a) (p-p65), Replicate 3. Target protein and β-actin were detected on the same membrane using the same protein lysates. The images show the full membranes from an independent biological replicate used for quantitative densitometric analysis presented in Figure 5 (a). All lanes are shown. No brightness or contrast adjustments were applied to individual bands.

Supplementary Figure S4. p65(Replicate1)

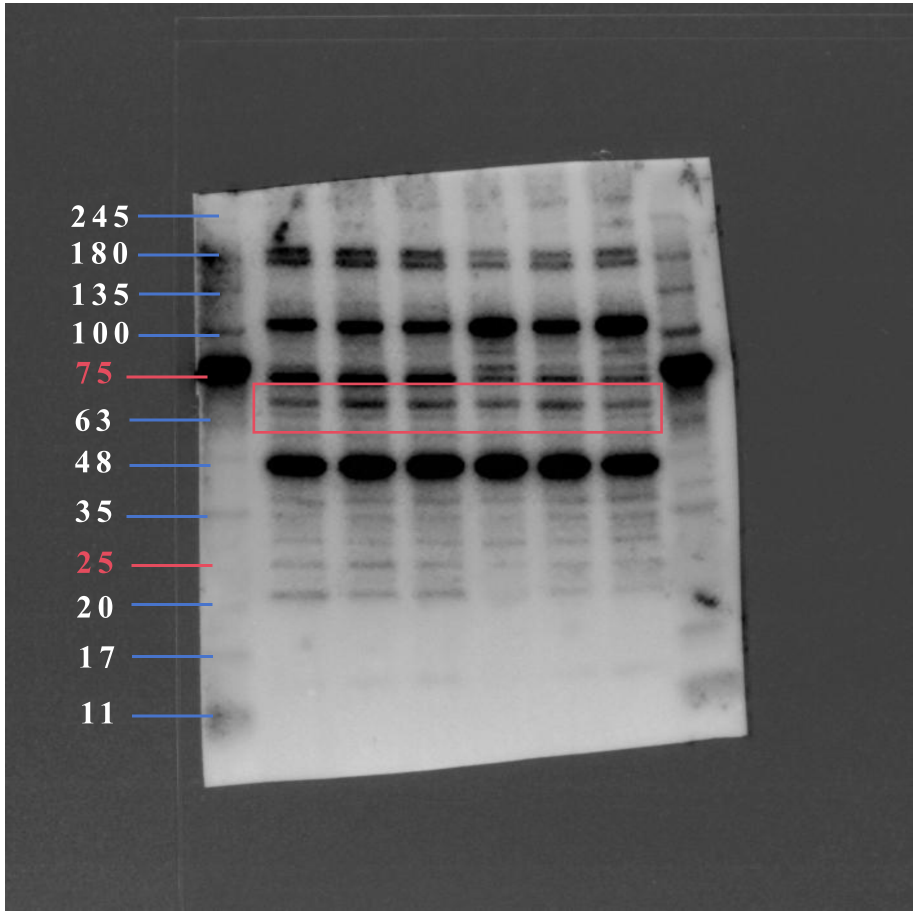

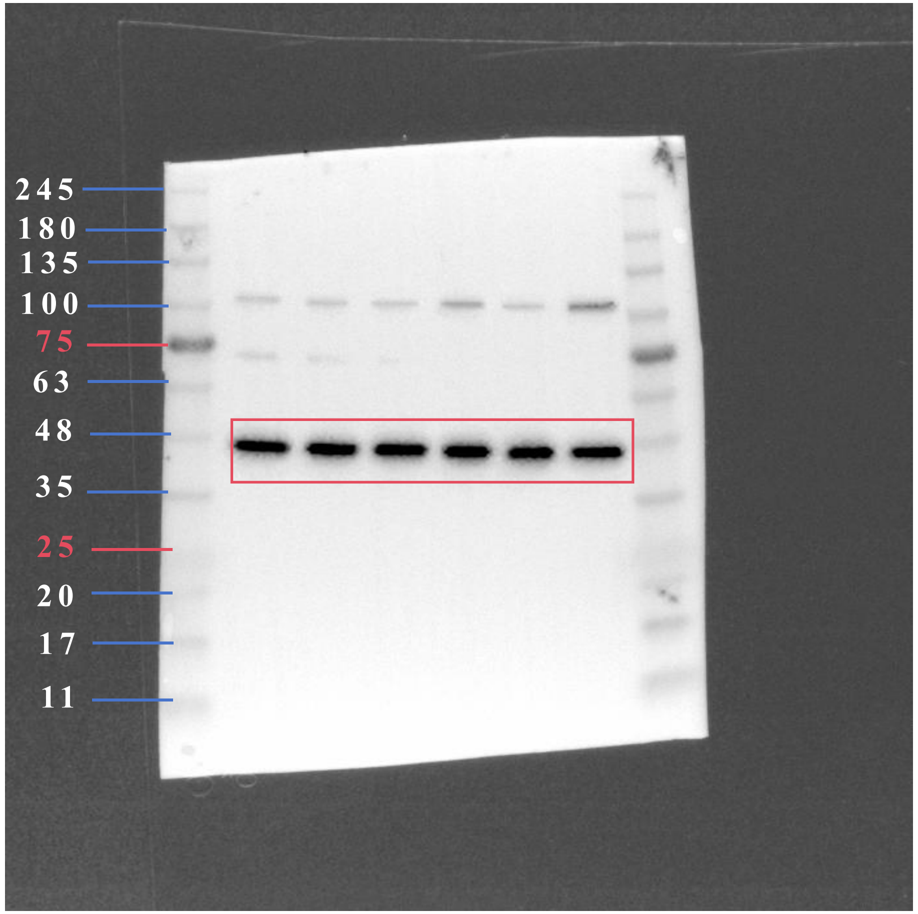

Lane1: Control

Lane2: BOT

Lane3: BAY11-7085

Lane4: TNF-α

Lane5: TNF-α+BOT

Lane6: TNF-α+BAY11-7085

Supplementary Figure S4.

Uncropped Western blot corresponding to Figure 5 (b), (p65), Replicate 1.Target protein and β-actin were detected on the same membrane using the same protein lysates. The images show the full membranes used to generate the main figure. Red boxes indicate the cropped regions presented in the manuscript. All lanes are shown. No brightness or contrast adjustments were applied to individual bands.

Supplementary Figure S5. p65(Replicate2)

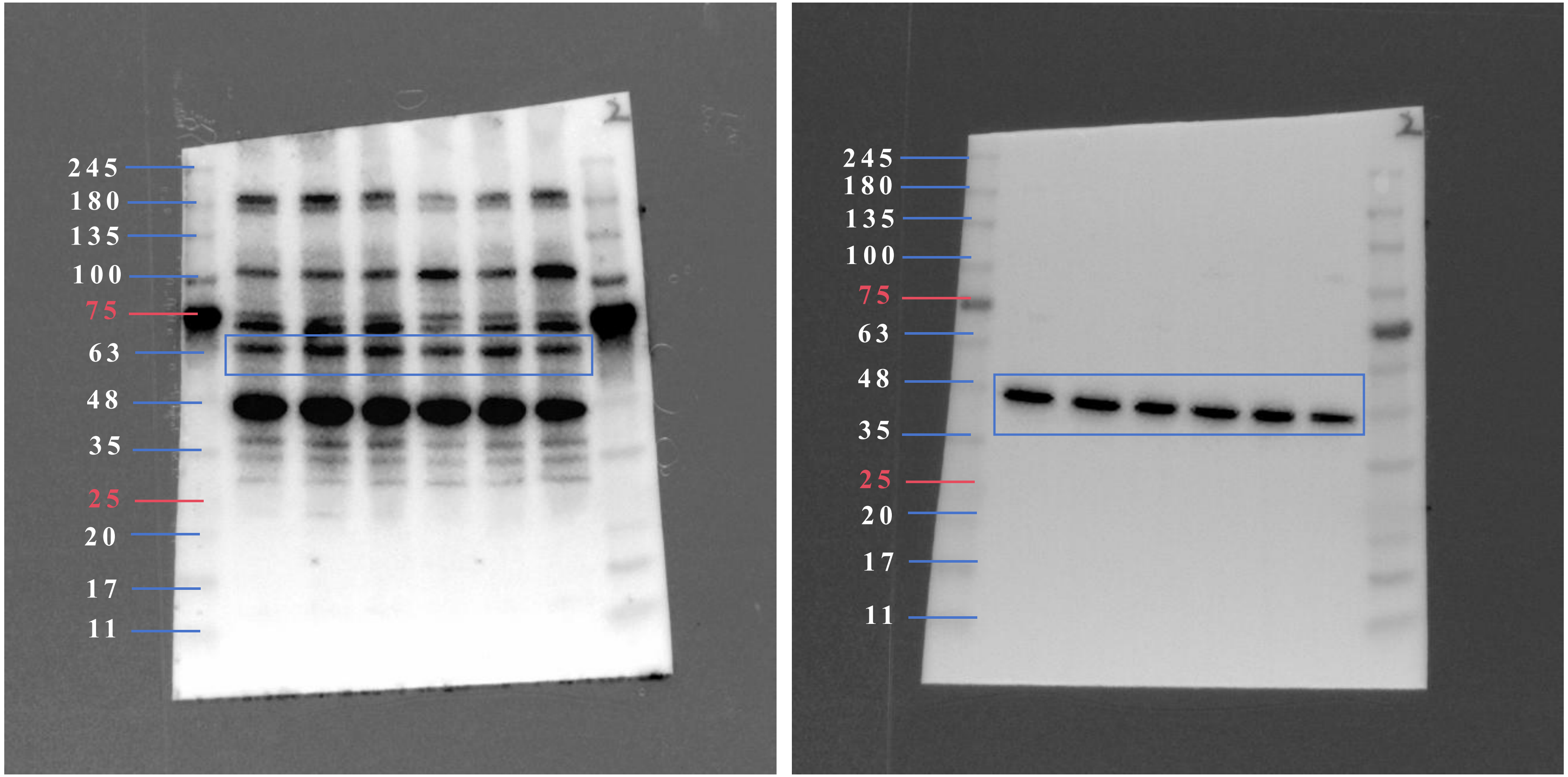

Lane1: Control

Lane2: BOT

Lane3: BAY11-7085

Lane4: TNF-α

Lane5: TNF-α+BOT

Lane6: TNF-α+BAY11-7085

Supplementary Figure S5.
Uncropped Western blot corresponding to Figure 5 (b) (p65), Replicate 2. Target protein and β-actin were detected on the same membrane using the same protein lysates. The images show the full membranes from an independent biological replicate used for quantitative densitometric analysis presented in Figure 5 (b). All lanes are shown. No brightness or contrast adjustments were applied to individual bands.

Supplementary Figure S6. p65(Replicate3)

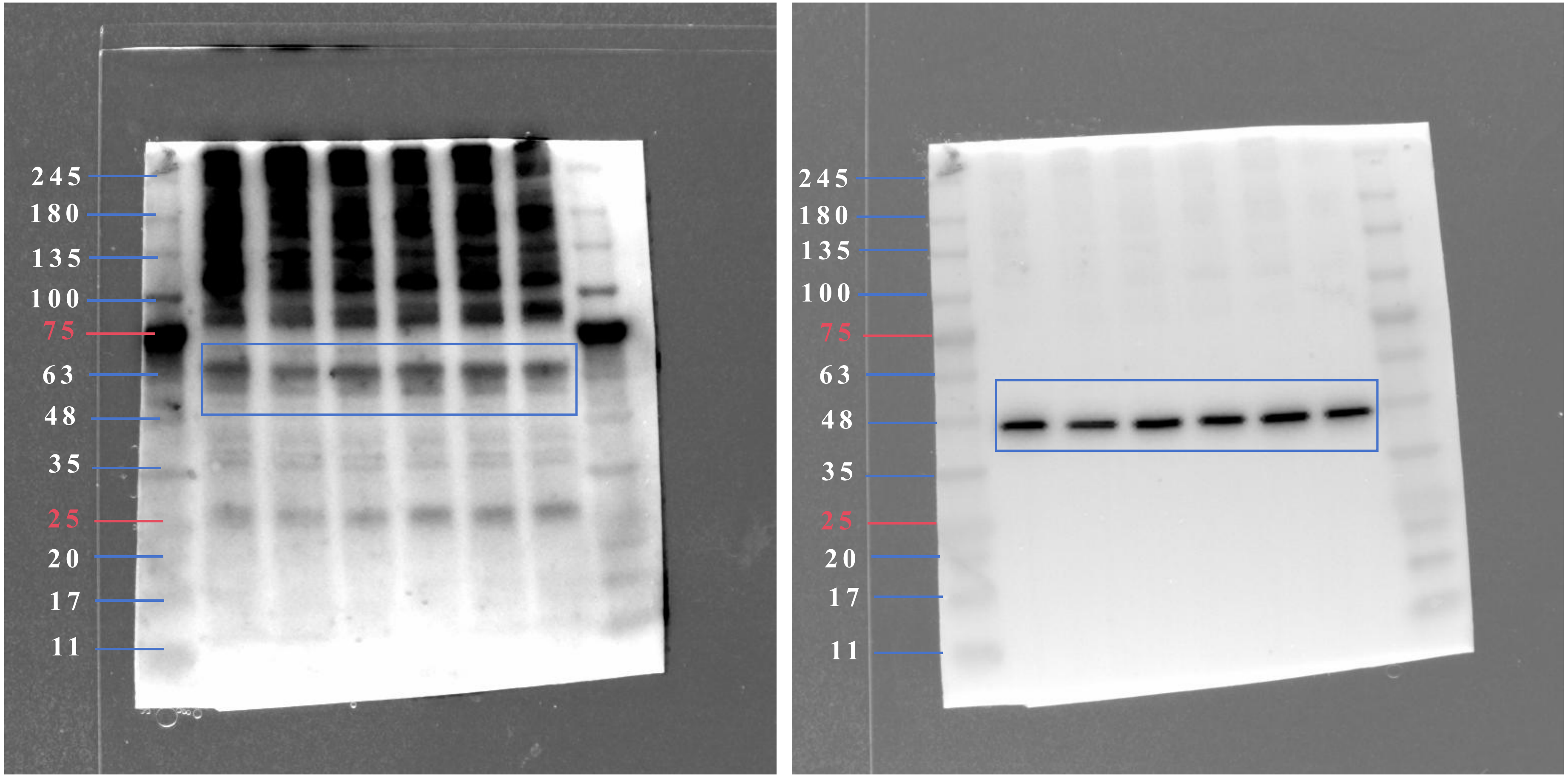

Lane1: Control

Lane2: BOT

Lane3: BAY11-7085

Lane4: TNF-α

Lane5: TNF-α+BOT

Lane6: TNF-α+BAY11-7085

Supplementary Figure S6.
Uncropped Western blot corresponding to Figure 5 (b) (p65), Replicate 3. Target protein and β-actin were detected on the same membrane using the same protein lysates. The images show the full membranes from an independent biological replicate used for quantitative densitometric analysis presented in Figure 5 (b). All lanes are shown. No brightness or contrast adjustments were applied to individual bands.

Supplementary Figure S7. p-ikb(Replicate1)

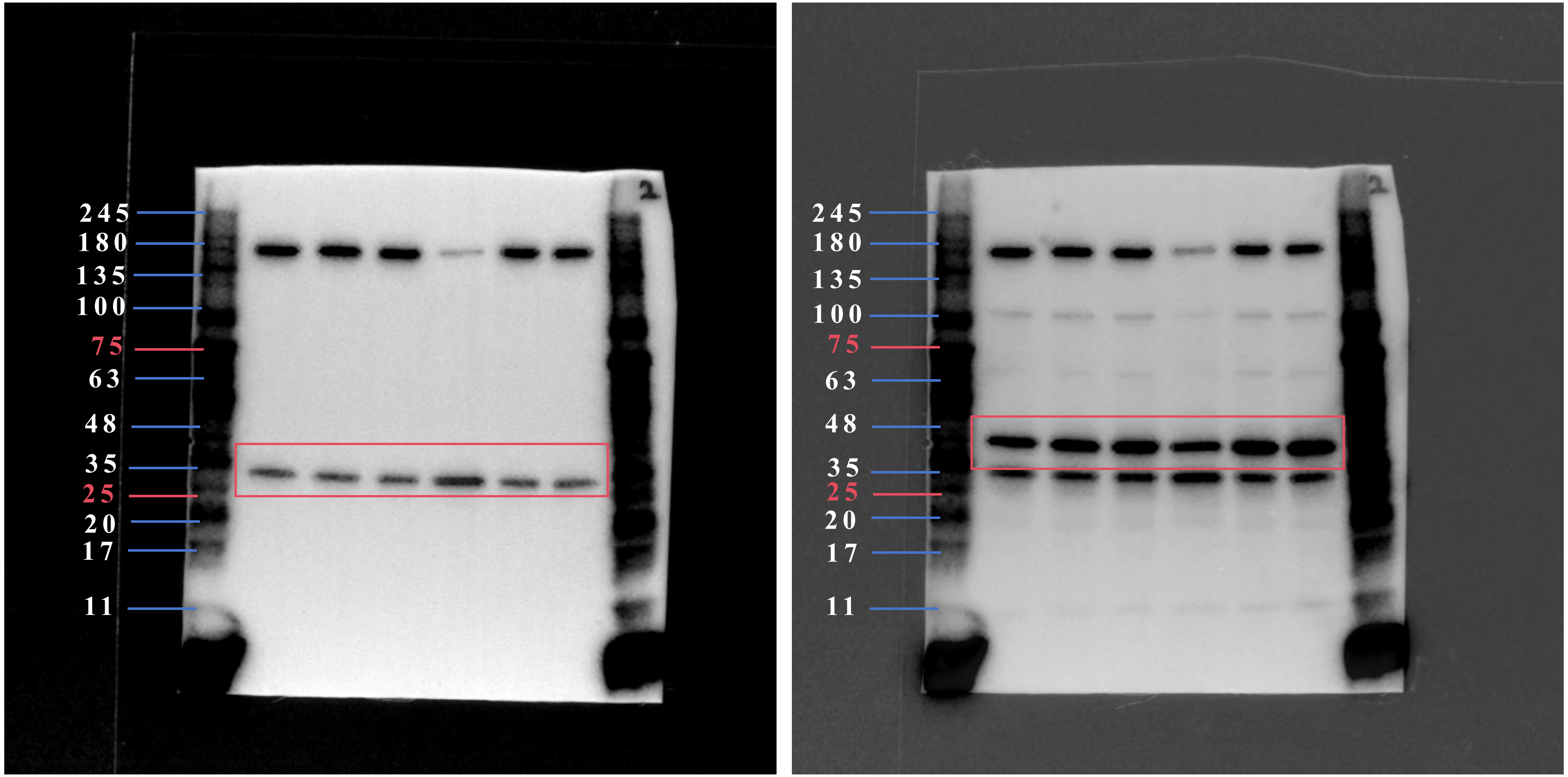

Lane1: Control

Lane2: BOT

Lane3: BAY11-7085

Lane4: TNF-α

Lane5: TNF-α+BOT

Lane6: TNF-α+BAY11-7085

Supplementary Figure S7.

Uncropped Western blot corresponding to Figure 5 (d), (p-ikb), Replicate 1.Target protein and β-actin were detected on the same membrane using the same protein lysates. The images show the full membranes used to generate the main figure. Red boxes indicate the cropped regions presented in the manuscript. All lanes are shown. No brightness or contrast adjustments were applied to individual bands.

Supplementary Figure S8. p-ikb(Replicate2)

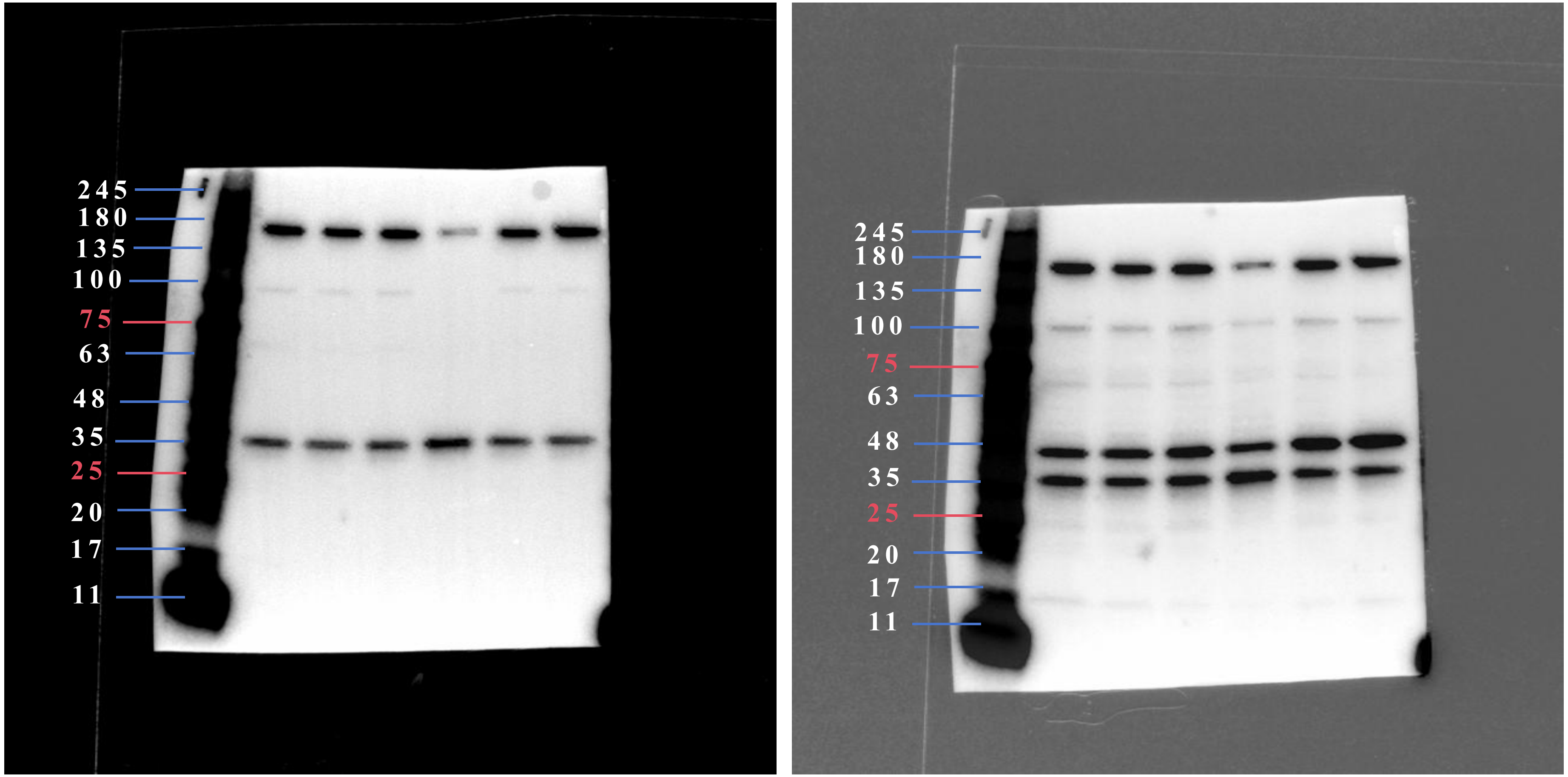

Lane1: Control

Lane2: BOT

Lane3: BAY11-7085

Lane4: TNF-α

Lane5: TNF-α+BOT

Lane6: TNF-α+BAY11-7085

Supplementary Figure S8.
Uncropped Western blot corresponding to Figure 5 (d) (p-ikb), Replicate 2. Target protein and β-actin were detected on the same membrane using the same protein lysates. The images show the full membranes from an independent biological replicate used for quantitative densitometric analysis presented in Figure 5 (d). All lanes are shown. No brightness or contrast adjustments were applied to individual bands.

Supplementary Figure S9. p-ikb(Replicate3)

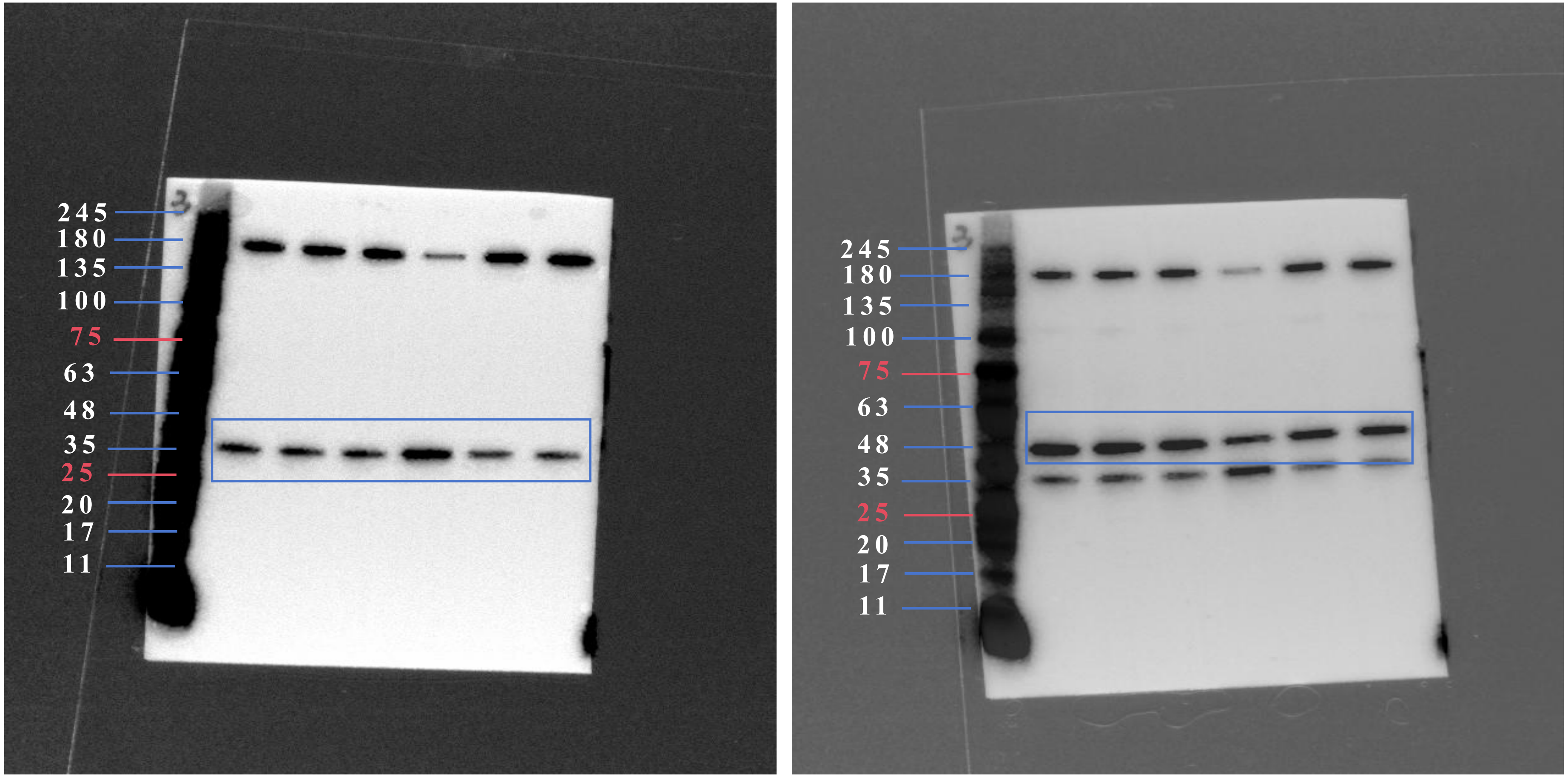

Lane1: Control

Lane2: BOT

Lane3: BAY11-7085

Lane4: TNF-α

Lane5: TNF-α+BOT

Lane6: TNF-α+BAY11-7085

Supplementary Figure S9.
Uncropped Western blot corresponding to Figure 5(d) (p-ikb), Replicate 3. Target protein and β-actin were detected on the same membrane using the same protein lysates. The images show the full membranes from an independent biological replicate used for quantitative densitometric analysis presented in Figure 5 (d). All lanes are shown. No brightness or contrast adjustments were applied to individual bands.

Supplementary Figure S10. ikb(Replicate1)

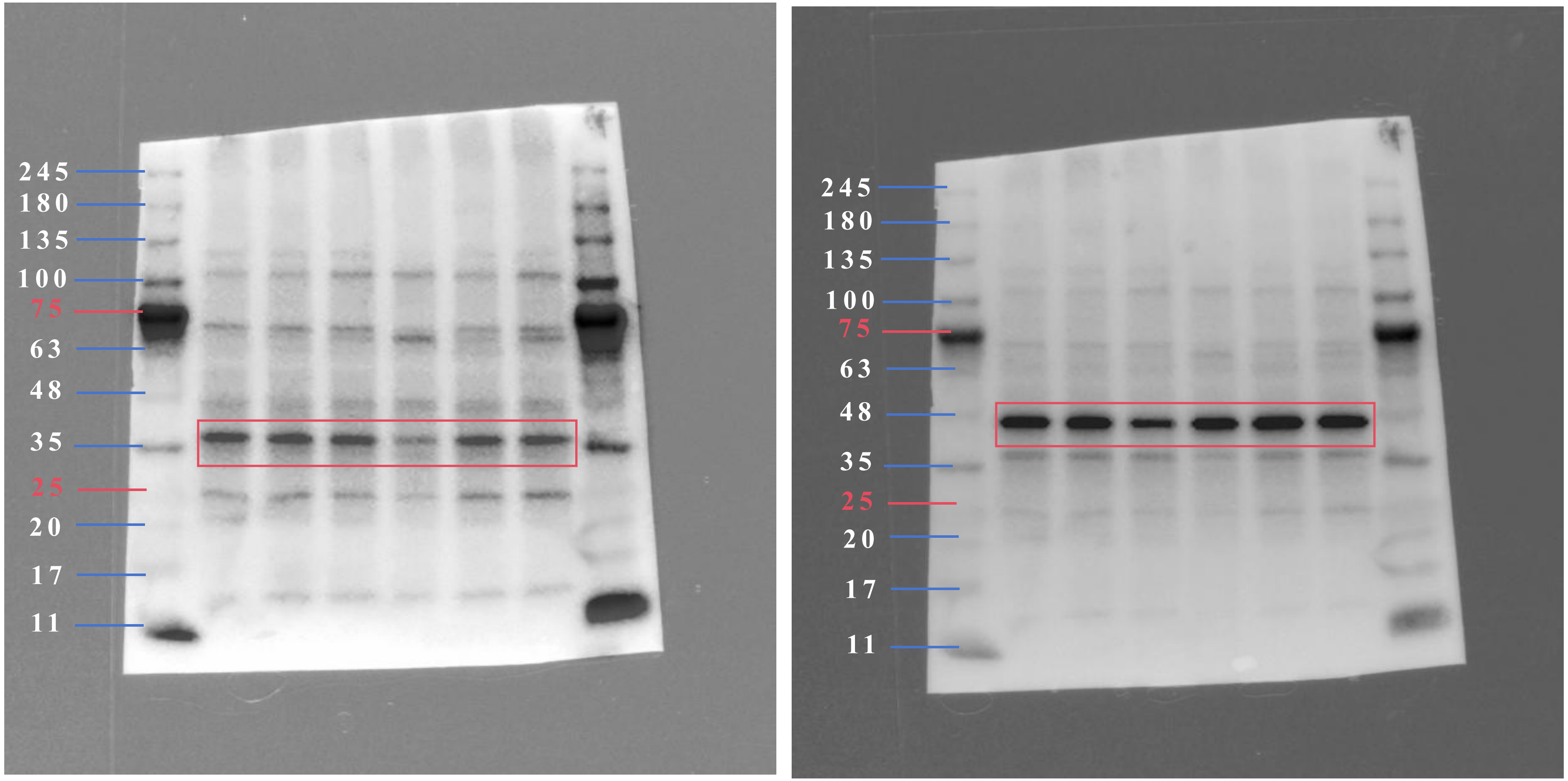

Lane1: Control

Lane2: BOT

Lane3: BAY11-7085

Lane4: TNF-α

Lane5: TNF-α+BOT

Lane6: TNF-α+BAY11-7085

Supplementary Figure S10.
Uncropped Western blot corresponding to Figure 5 (e), (ikb), Replicate 1.Target protein and β-actin were detected on the same membrane using the same protein lysates. The images show the full membranes used to generate the main figure. Red boxes indicate the cropped regions presented in the manuscript. All lanes are shown. No brightness or contrast adjustments were applied to individual bands.

Supplementary Figure S11. ikb(Replicate2)

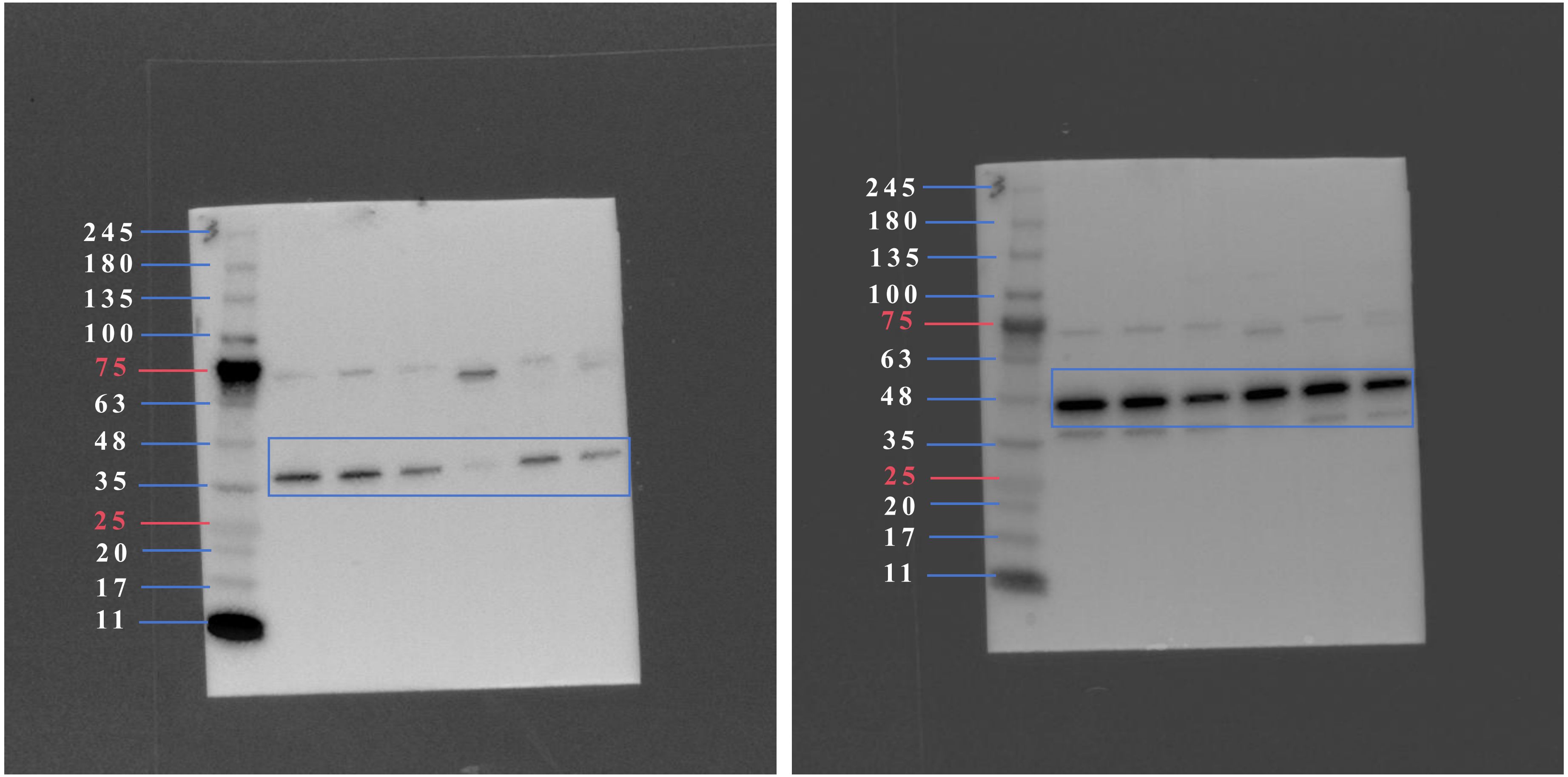

Lane1: Control

Lane2: BOT

Lane3: BAY11-7085

Lane4: TNF-α

Lane5: TNF-α+BOT

Lane6: TNF-α+BAY11-7085

Supplementary Figure S11.
Uncropped Western blot corresponding to Figure 5 (e) (p-p65), Replicate 2. Target protein and β-actin were detected on the same membrane using the same protein lysates. The images show the full membranes from an independent biological replicate used for quantitative densitometric analysis presented in Figure 5 (e). All lanes are shown. No brightness or contrast adjustments were applied to individual bands.

Supplementary Figure S12. ikb(Replicate3)

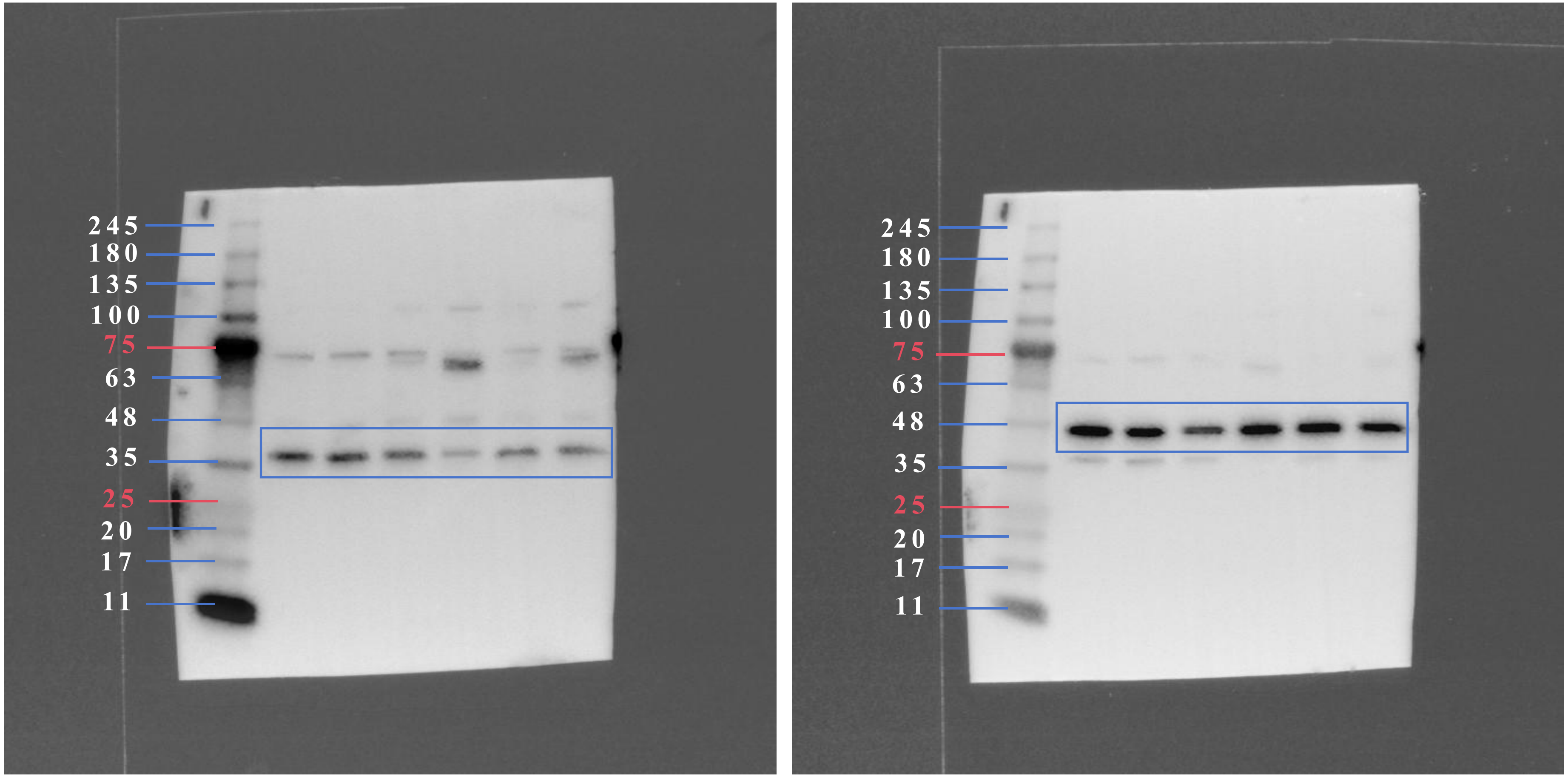

Lane1: Control

Lane2: BOT

Lane3: BAY11-7085

Lane4: TNF-α

Lane5: TNF-α+BOT

Lane6: TNF-α+BAY11-7085

Supplementary Figure S12.
Uncropped Western blot corresponding to Figure 5 (e) (ikb), Replicate 3. Target protein and β-actin were detected on the same membrane using the same protein lysates. The images show the full membranes from an independent biological replicate used for quantitative densitometric analysis presented in Figure 5 (e). All lanes are shown. No brightness or contrast adjustments were applied to individual bands.

Supplementary Figure S13. p-p65(Replicate1)

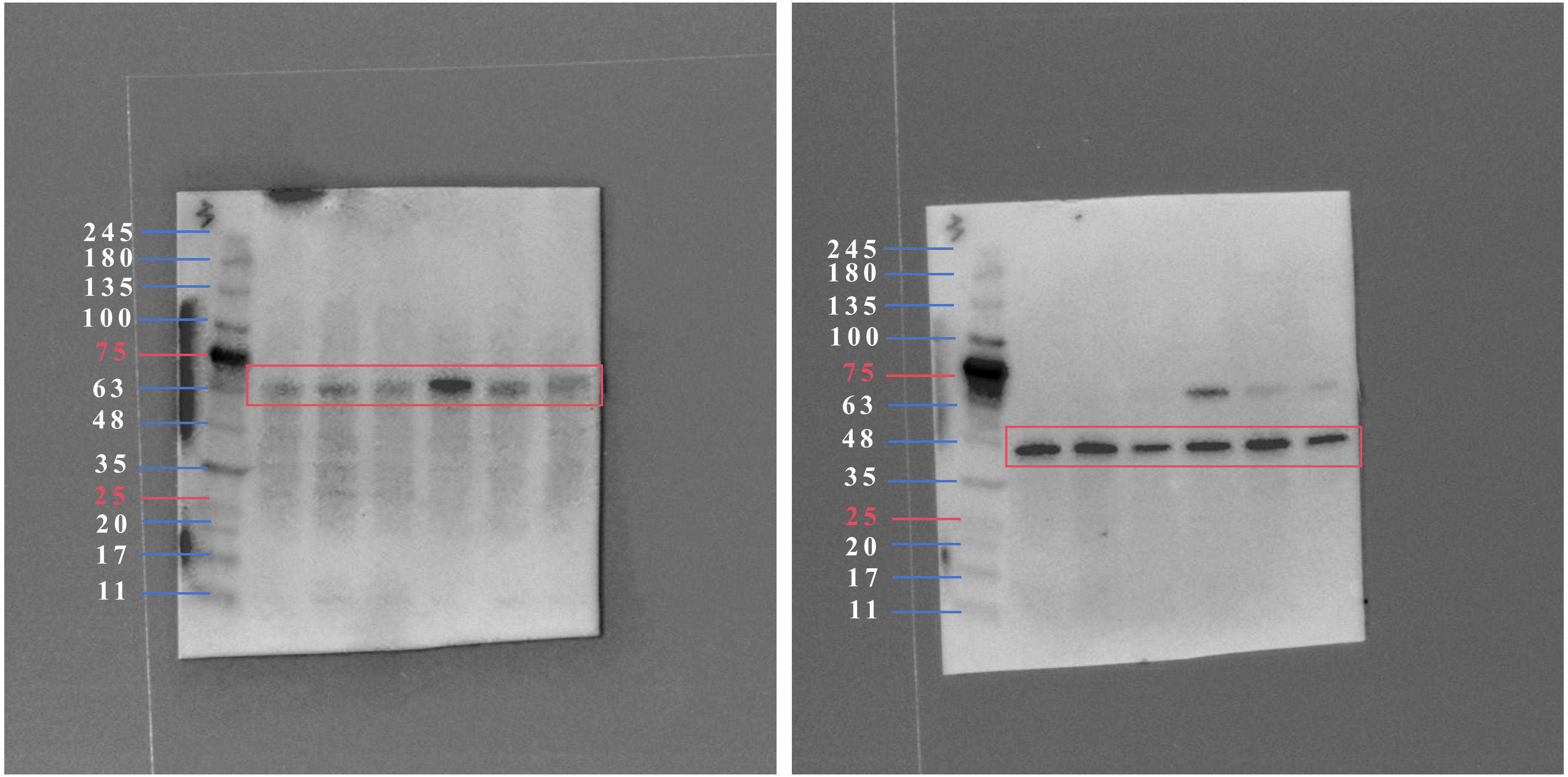

Lane1: Control

Lane2: BOT

Lane3: BAY11-7085

Lane4: TNF-α

Lane5: TNF-α+BOT

Lane6: TNF-α+BAY11-7085

Supplementary Figure S13.
Uncropped Western blot corresponding to Figure 9 (a), (p-p65), Replicate 1.Target protein and β-actin were detected on the same membrane using the same protein lysates. The images show the full membranes used to generate the main figure. Red boxes indicate the cropped regions presented in the manuscript. All lanes are shown. No brightness or contrast adjustments were applied to individual bands.

Supplementary Figure S14. p-p65(Replicate2)

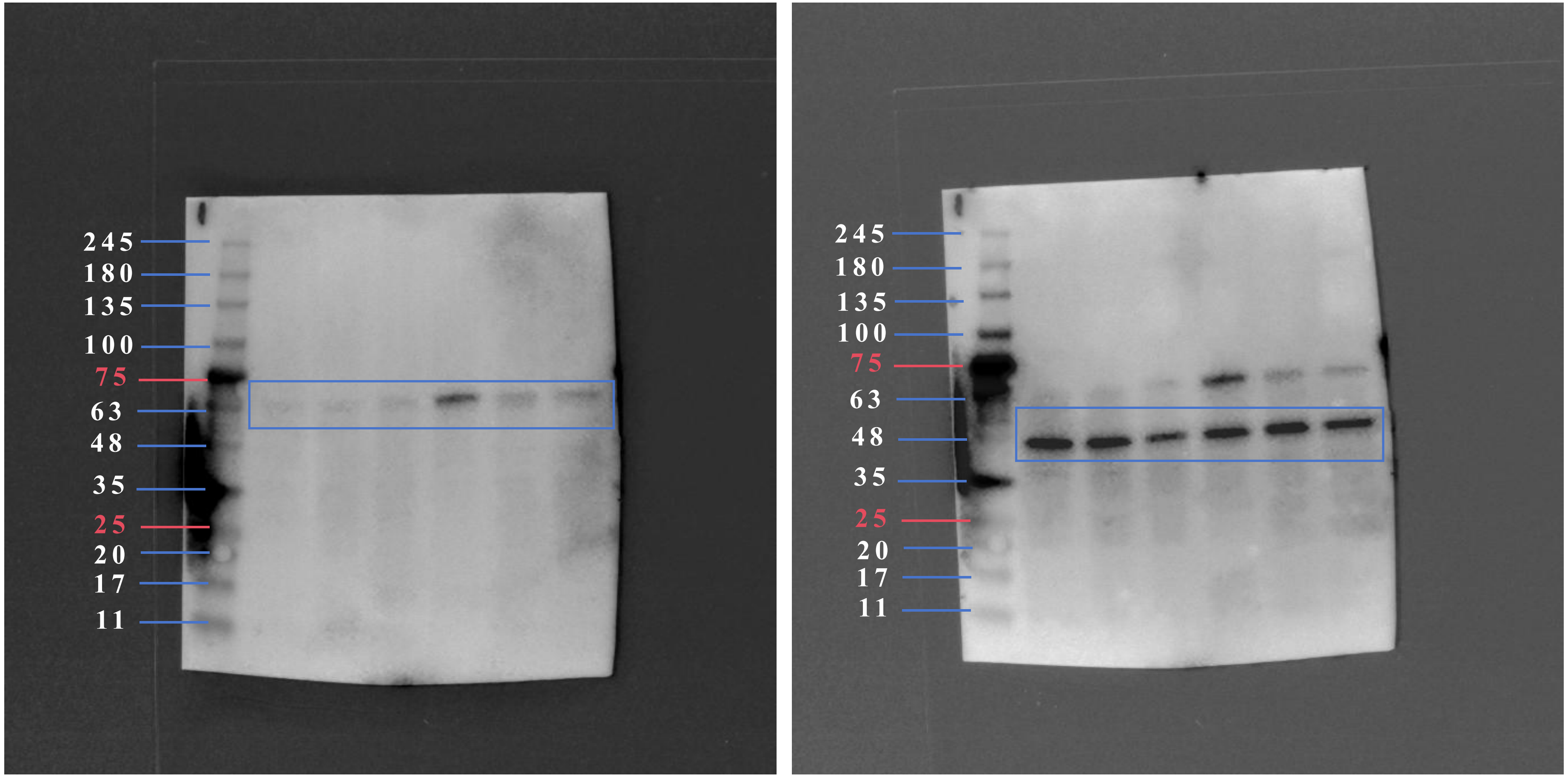

Lane1: Control

Lane2: BOT

Lane3: BAY11-7085

Lane4: TNF-α

Lane5: TNF-α+BOT

Lane6: TNF-α+BAY11-7085

Supplementary Figure S14.
Uncropped Western blot corresponding to Figure 9 (a) (p-p65), Replicate 2. Target protein and β-actin were detected on the same membrane using the same protein lysates. The images show the full membranes from an independent biological replicate used for quantitative densitometric analysis presented in Figure 9 (a). All lanes are shown. No brightness or contrast adjustments were applied to individual bands.

Supplementary Figure S15. p-p65(Replicate3)

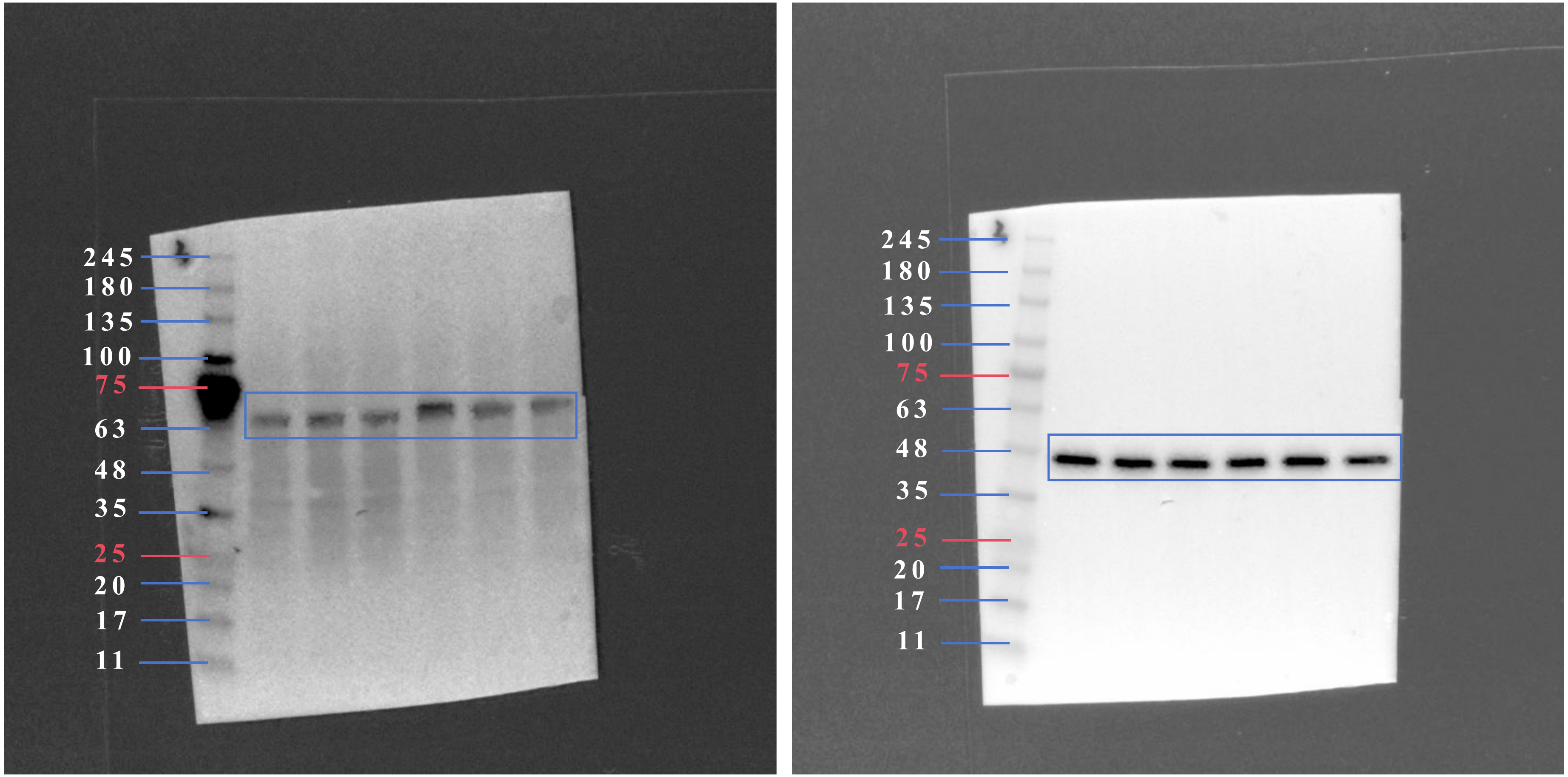

Lane1: Control

Lane2: BOT

Lane3: BAY11-7085

Lane4: TNF-α

Lane5: TNF-α+BOT

Lane6: TNF-α+BAY11-7085

Supplementary Figure S15.
Uncropped Western blot corresponding to Figure 9 (a) (p-p65), Replicate 3. Target protein and β-actin were detected on the same membrane using the same protein lysates. The images show the full membranes from an independent biological replicate used for quantitative densitometric analysis presented in Figure 9 (a). All lanes are shown. No brightness or contrast adjustments were applied to individual bands.

Supplementary Figure S16. p65(Replicate1)

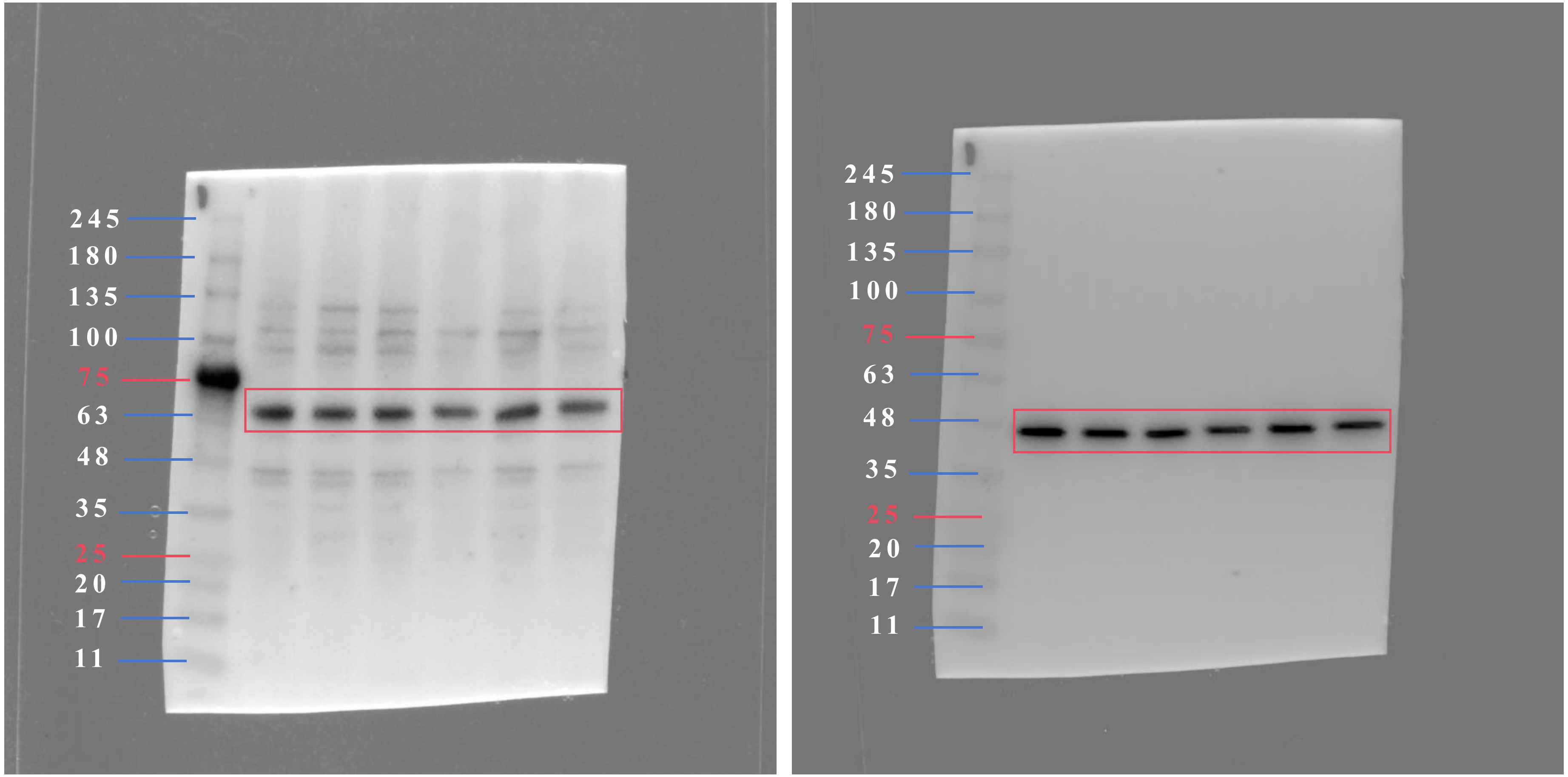

Lane1: Control

Lane2: BOT

Lane3: BAY11-7085

Lane4: TNF-α

Lane5: TNF-α+BOT

Lane6: TNF-α+BAY11-7085

Supplementary Figure S16.
Uncropped Western blot corresponding to Figure 9 (b), (p65), Replicate 1.Target protein and β-actin were detected on the same membrane using the same protein lysates. The images show the full membranes used to generate the main figure. Red boxes indicate the cropped regions presented in the manuscript. All lanes are shown. No brightness or contrast adjustments were applied to individual bands.

Supplementary Figure S17. p65(Replicate2)

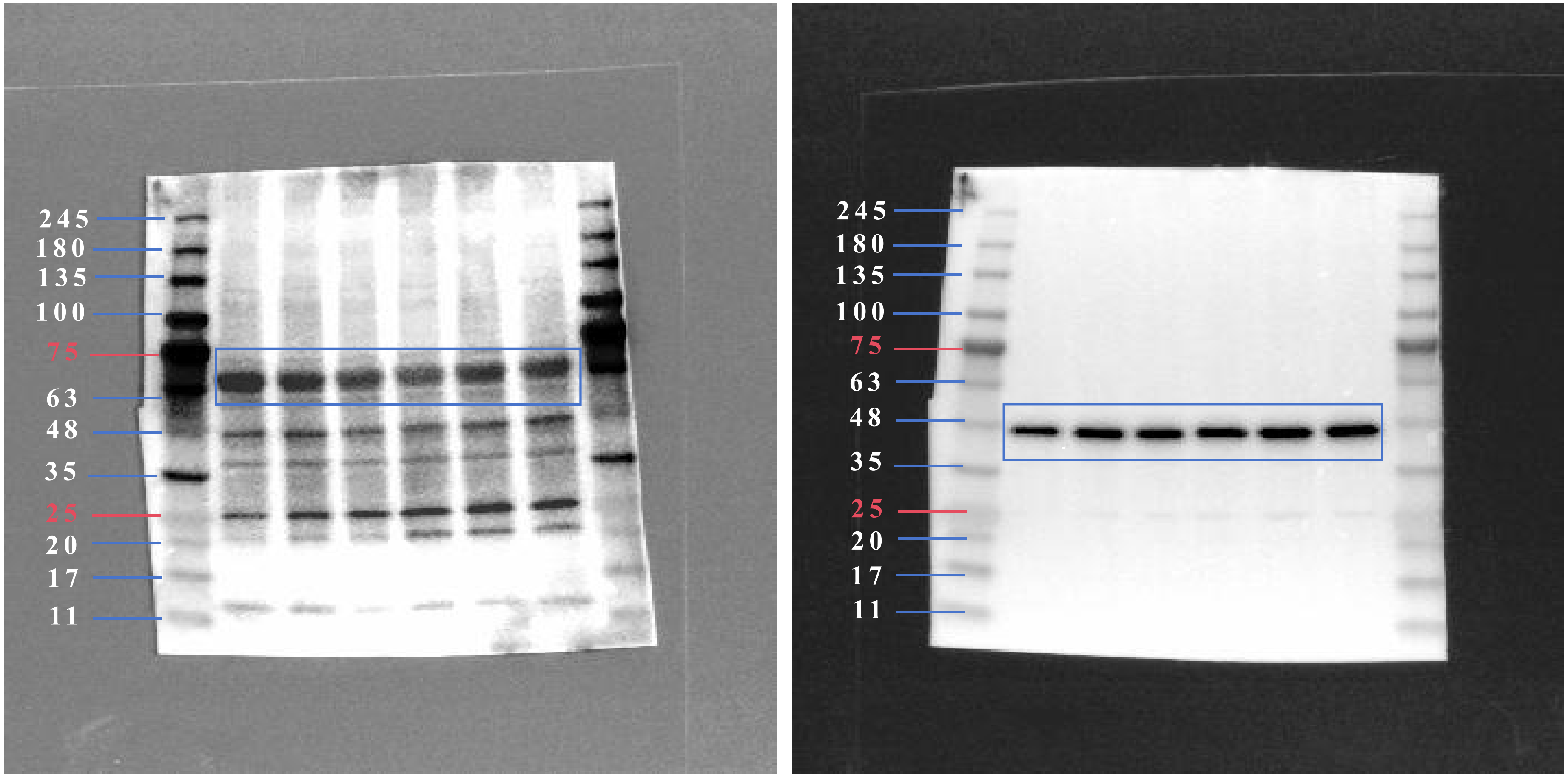

Lane1: Control

Lane2: BOT

Lane3: BAY11-7085

Lane4: TNF-α

Lane5: TNF-α+BOT

Lane6: TNF-α+BAY11-7085

Supplementary Figure S17.
Uncropped Western blot corresponding to Figure 9 (b) (p65), Replicate 2. Target protein and β-actin were detected on the same membrane using the same protein lysates. The images show the full membranes from an independent biological replicate used for quantitative densitometric analysis presented in Figure 9 (b). All lanes are shown. No brightness or contrast adjustments were applied to individual bands.

Supplementary Figure S18. p65(Replicate3)

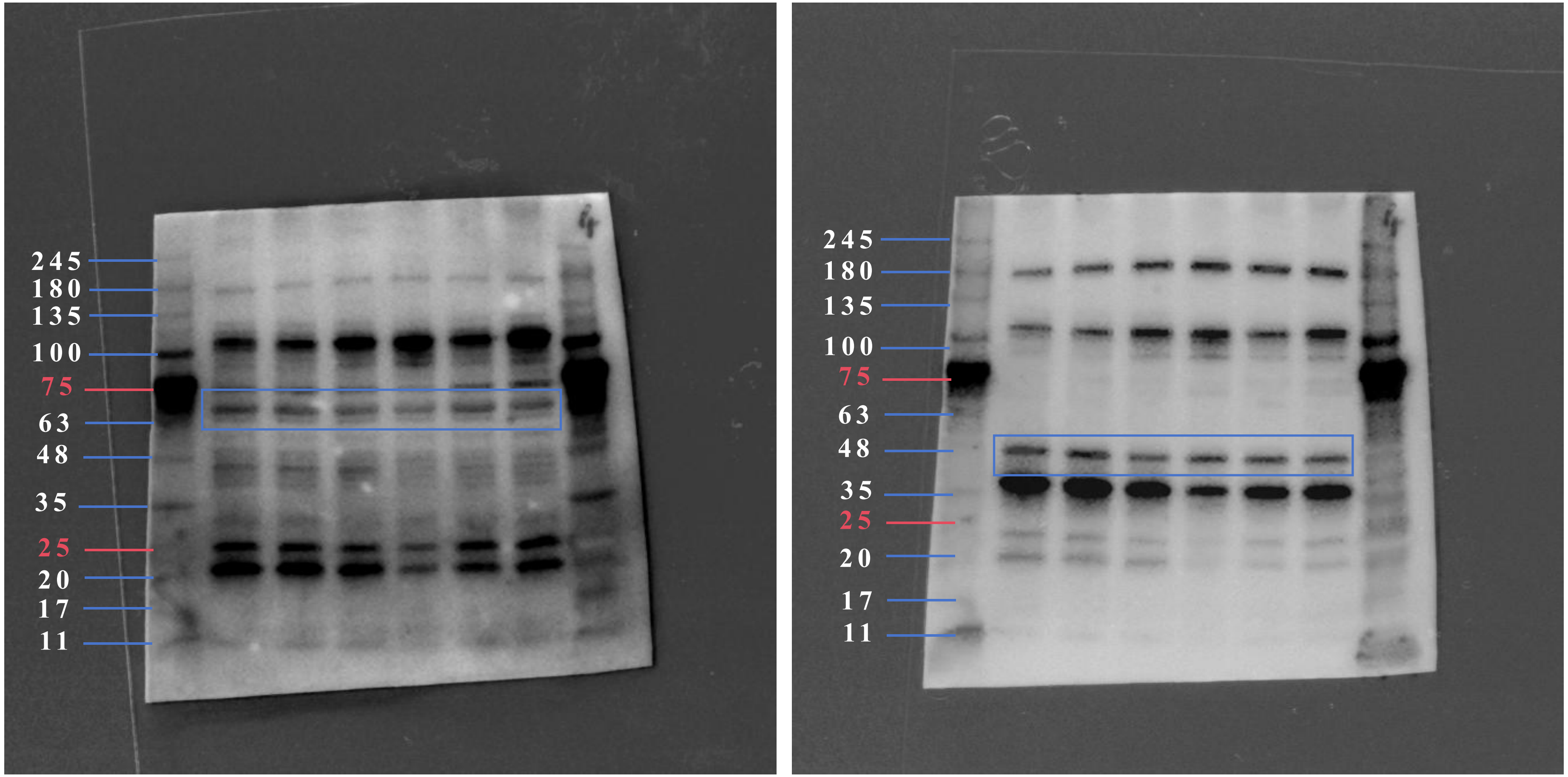

Lane1: Control

Lane2: BOT

Lane3: BAY11-7085

Lane4: TNF-α

Lane5: TNF-α+BOT

Lane6: TNF-α+BAY11-7085

Supplementary Figure S18.
Uncropped Western blot corresponding to Figure 9 (b) (p65), Replicate 3. Target protein and β-actin were detected on the same membrane using the same protein lysates. The images show the full membranes from an independent biological replicate used for quantitative densitometric analysis presented in Figure 9 (b). All lanes are shown. No brightness or contrast adjustments were applied to individual bands.

Supplementary Figure S19. p-ikb(Replicate1)

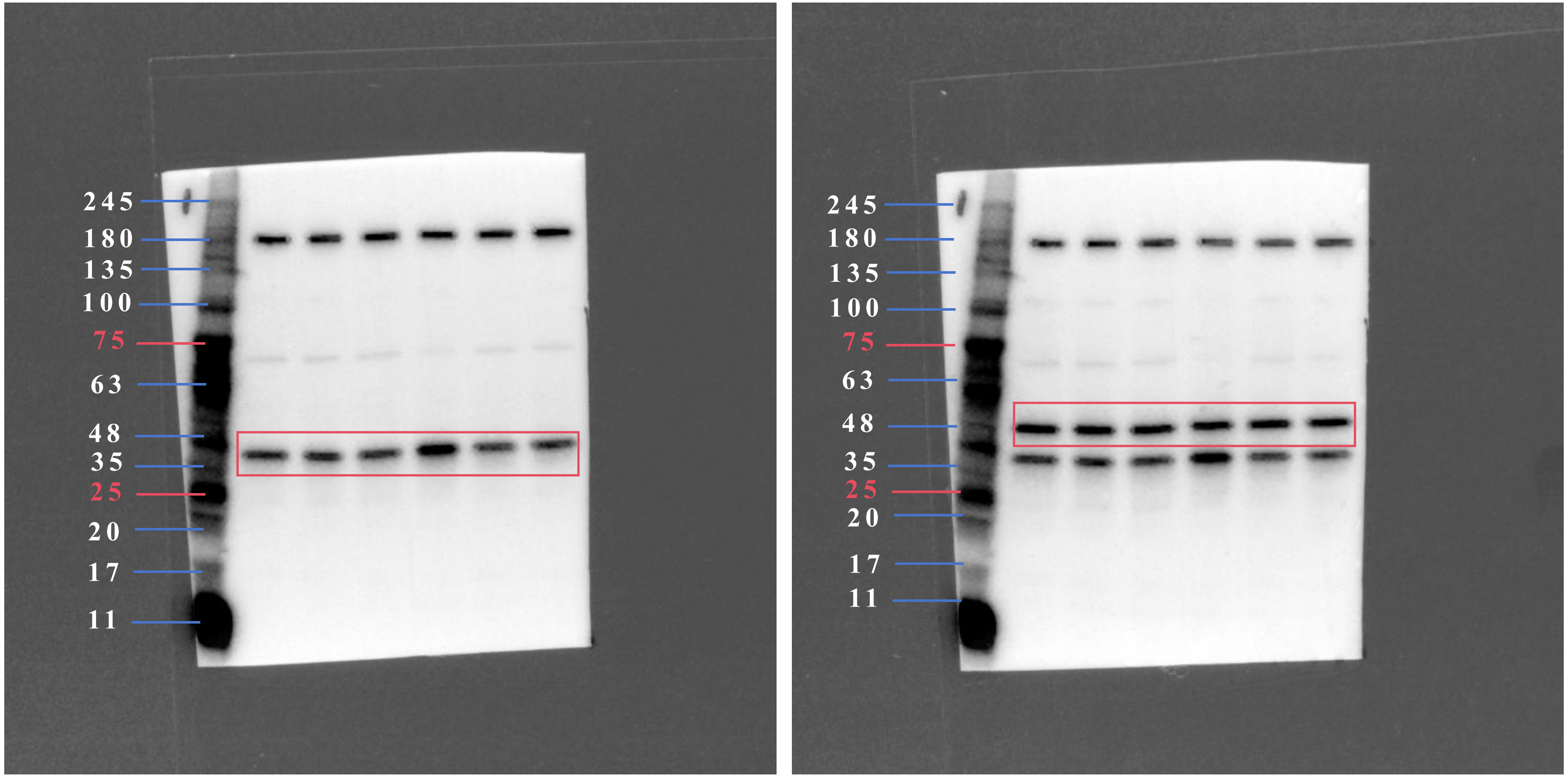

Lane1: Control

Lane2: BOT

Lane3: BAY11-7085

Lane4: TNF-α

Lane5: TNF-α+BOT

Lane6: TNF-α+BAY11-7085

Supplementary Figure S19.
Uncropped Western blot corresponding to Figure 9 (d), (p-ikb), Replicate 1.Target protein and β-actin were detected on the same membrane using the same protein lysates. The images show the full membranes used to generate the main figure. Red boxes indicate the cropped regions presented in the manuscript. All lanes are shown. No brightness or contrast adjustments were applied to individual bands.

Supplementary Figure S20. p-ikb(Replicate2)

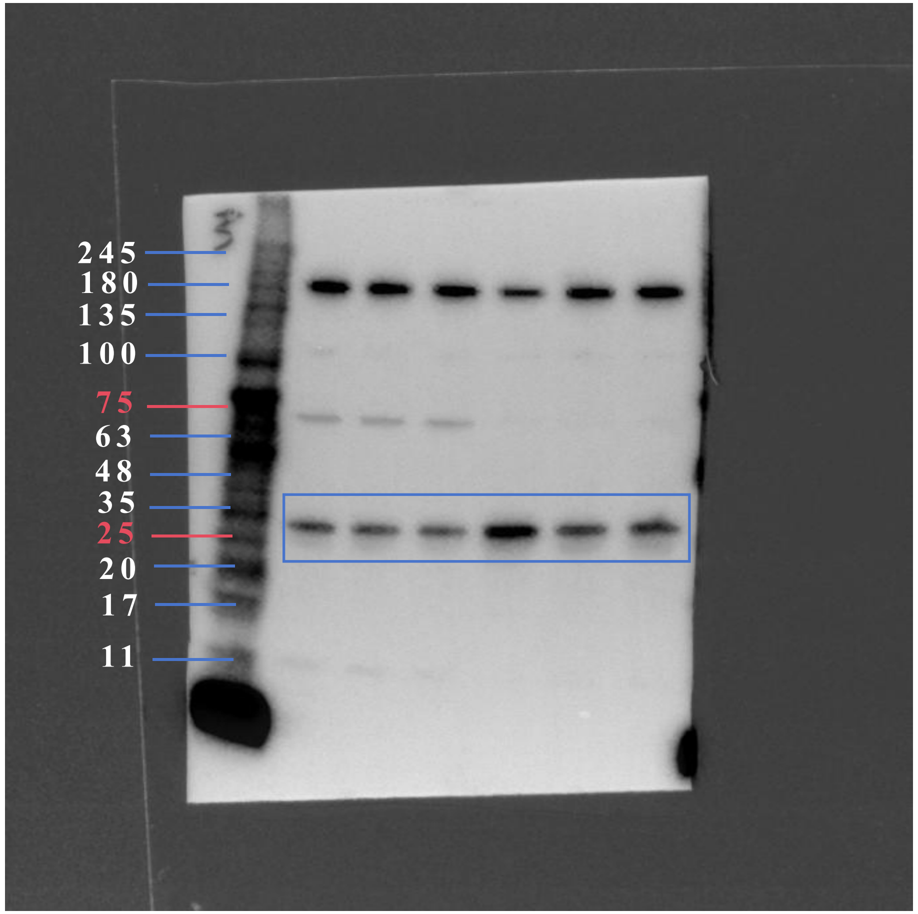

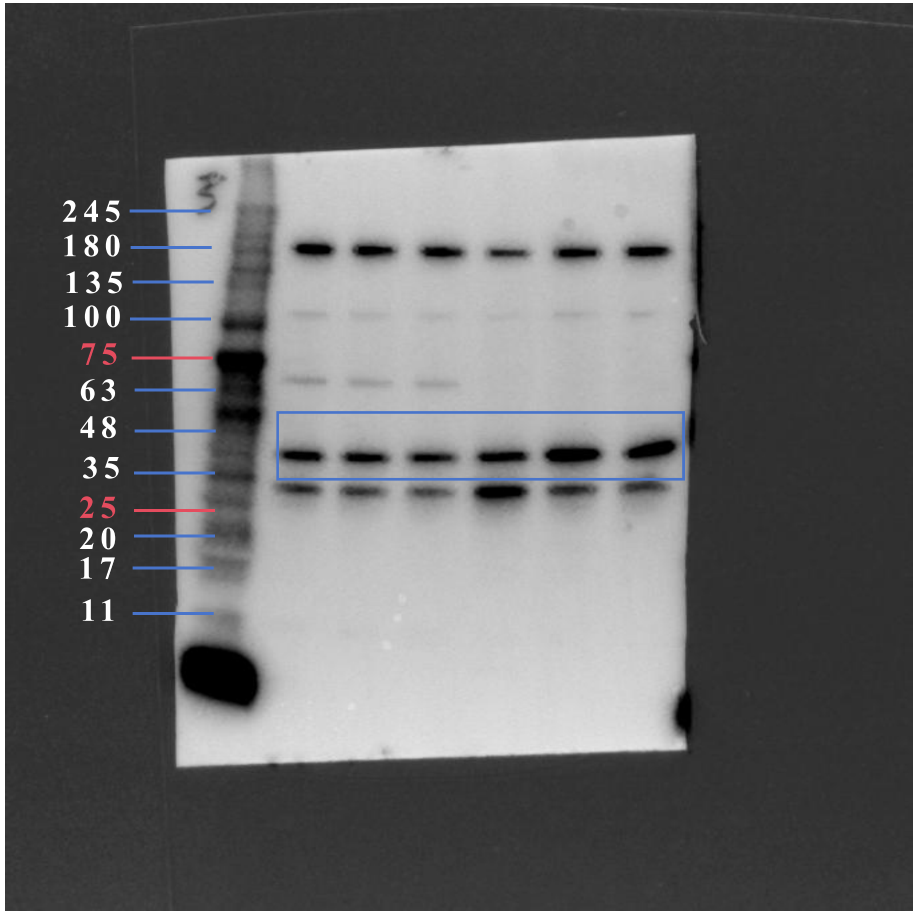

Lane1: Control

Lane2: BOT

Lane3: BAY11-7085

Lane4: TNF-α

Lane5: TNF-α+BOT

Lane6: TNF-α+BAY11-7085

Supplementary Figure S20.
Uncropped Western blot corresponding to Figure 9 (d) (p-ikb), Replicate 2. Target protein and β-actin were detected on the same membrane using the same protein lysates. The images show the full membranes from an independent biological replicate used for quantitative densitometric analysis presented in Figure 9 (d). All lanes are shown. No brightness or contrast adjustments were applied to individual bands.

Supplementary Figure S21. p-ikb(Replicate3)

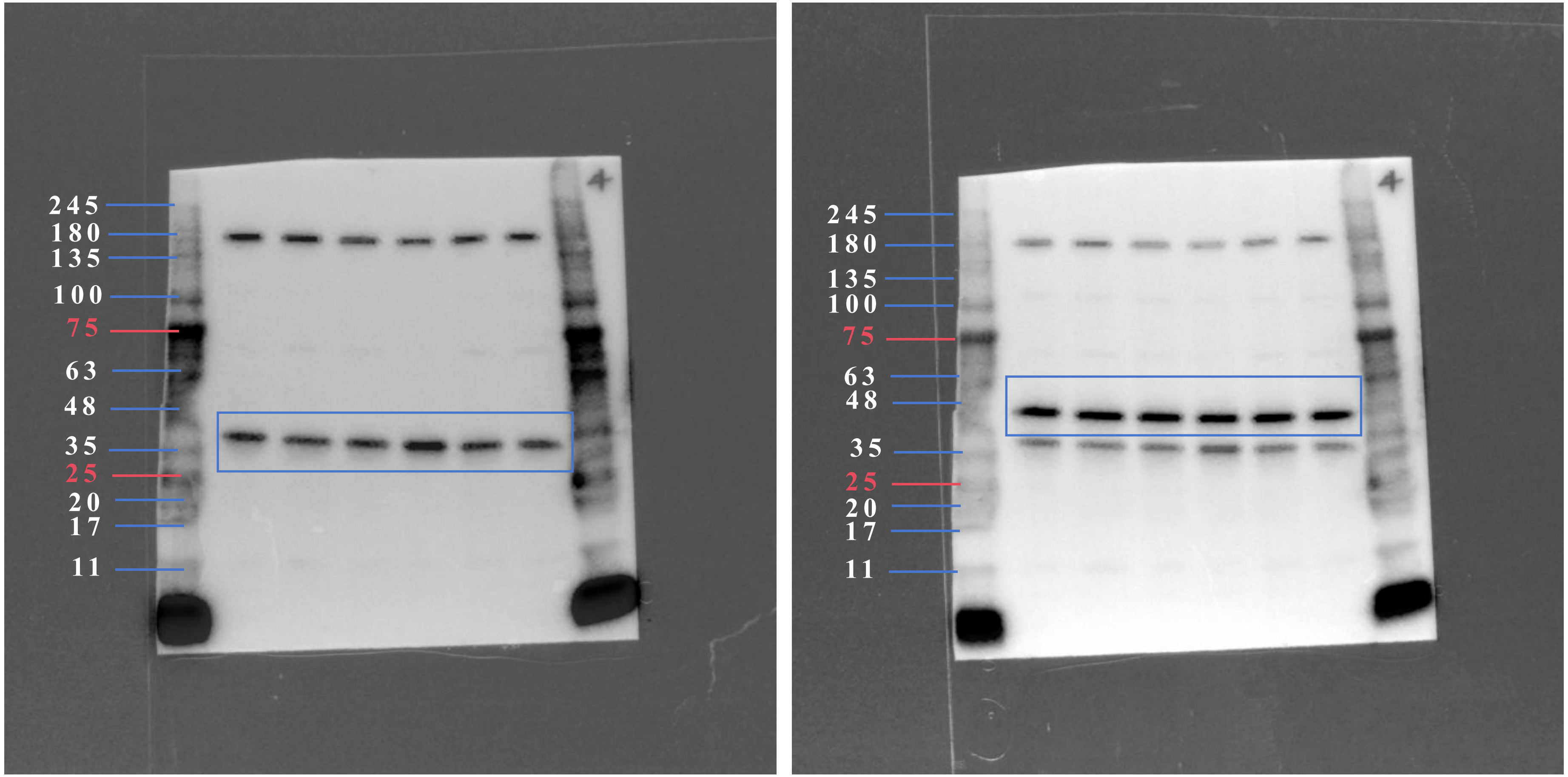

Lane1: Control

Lane2: BOT

Lane3: BAY11-7085

Lane4: TNF-α

Lane5: TNF-α+BOT

Lane6: TNF-α+BAY11-7085

Supplementary Figure S21.
Uncropped Western blot corresponding to Figure 9 (d) (p-ikb), Replicate 3. Target protein and β-actin were detected on the same membrane using the same protein lysates. The images show the full membranes from an independent biological replicate used for quantitative densitometric analysis presented in Figure 9 (d). All lanes are shown. No brightness or contrast adjustments were applied to individual bands.

Supplementary Figure S22. ikb(Replicate1)

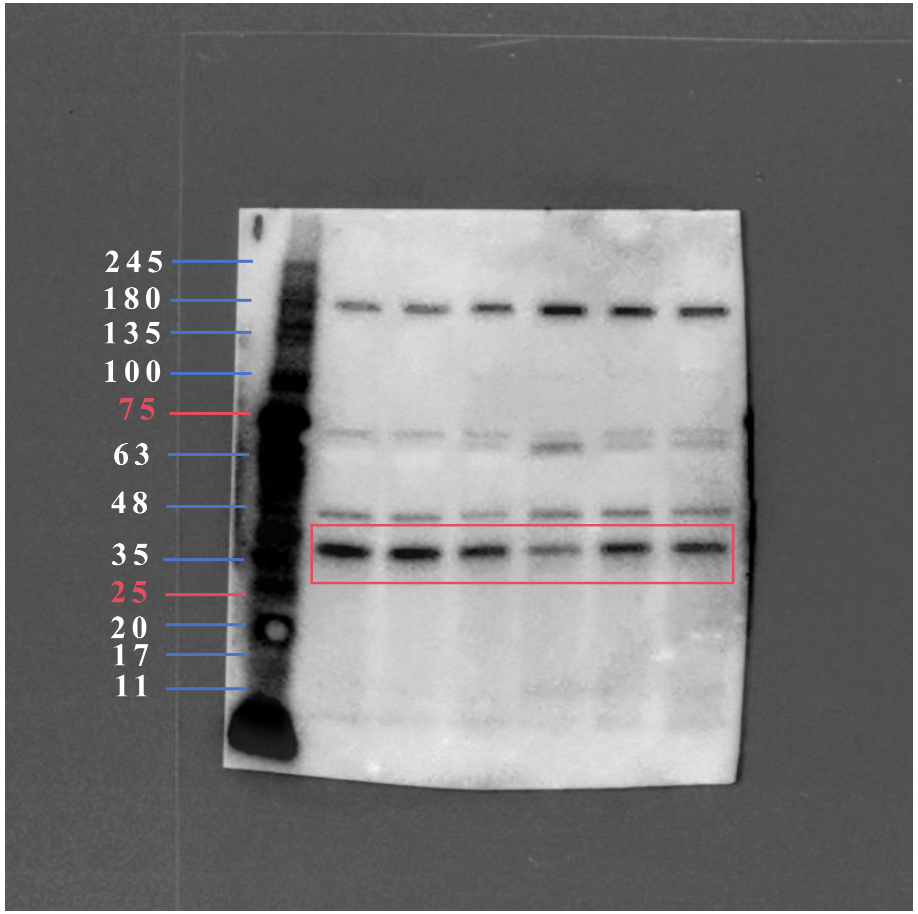

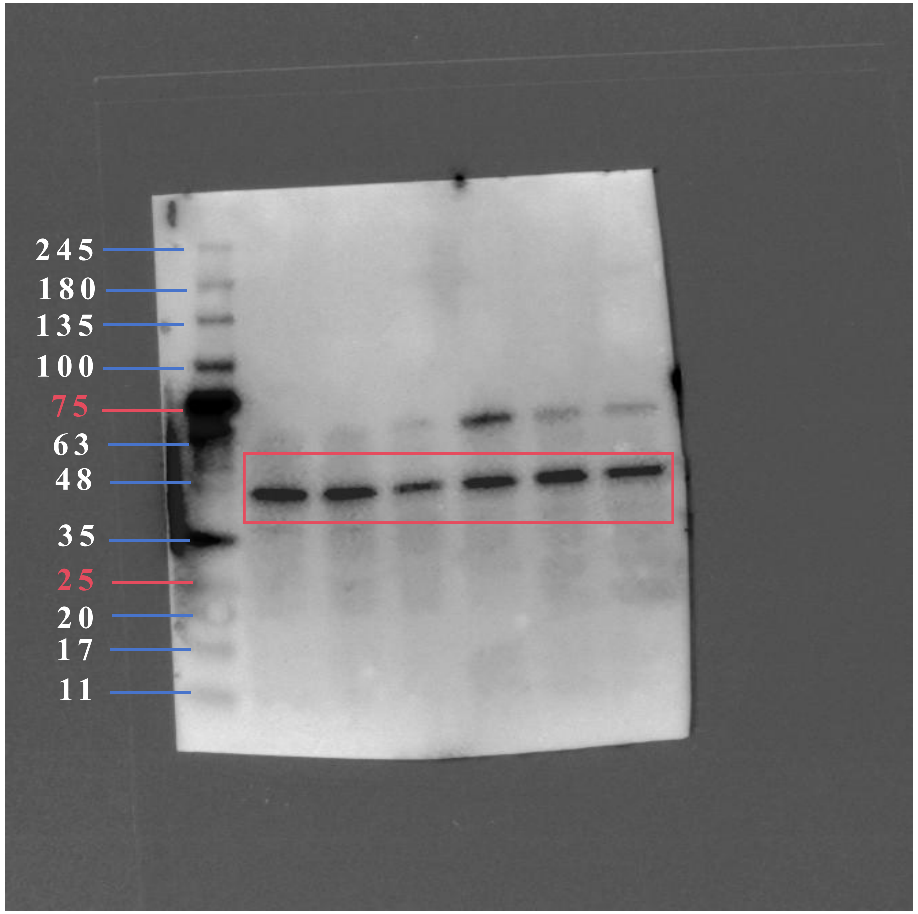

Lane1: Control

Lane2: BOT

Lane3: BAY11-7085

Lane4: TNF-α

Lane5: TNF-α+BOT

Lane6: TNF-α+BAY11-7085

Supplementary Figure S22.
Uncropped Western blot corresponding to Figure 9 (e), (ikb), Replicate 1.Target protein and β-actin were detected on the same membrane using the same protein lysates. The images show the full membranes used to generate the main figure. Red boxes indicate the cropped regions presented in the manuscript. All lanes are shown. No brightness or contrast adjustments were applied to individual bands.

Supplementary Figure S23. ikb(Replicate2)

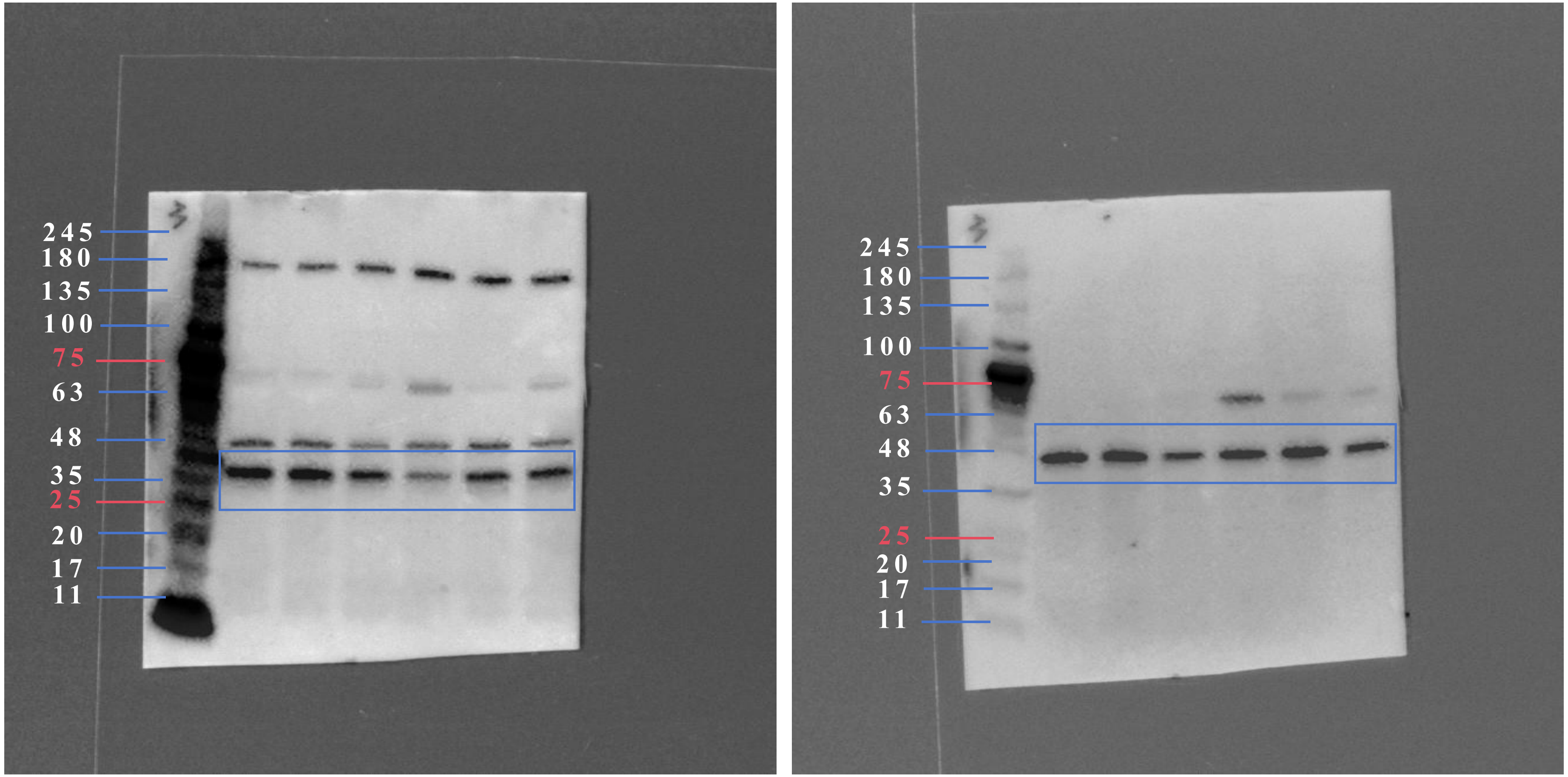

Lane1: Control

Lane2: BOT

Lane3: BAY11-7085

Lane4: TNF-α

Lane5: TNF-α+BOT

Lane6: TNF-α+BAY11-7085

Supplementary Figure S23.

Uncropped Western blot corresponding to Figure 9 (e) (ikb), Replicate 2. Target protein and β-actin were detected on the same membrane using the same protein lysates. The images show the full membranes from an independent biological replicate used for quantitative densitometric analysis presented in Figure 9 (e). All lanes are shown. No brightness or contrast adjustments were applied to individual bands.

Supplementary Figure S24. ikb(Replicate3)

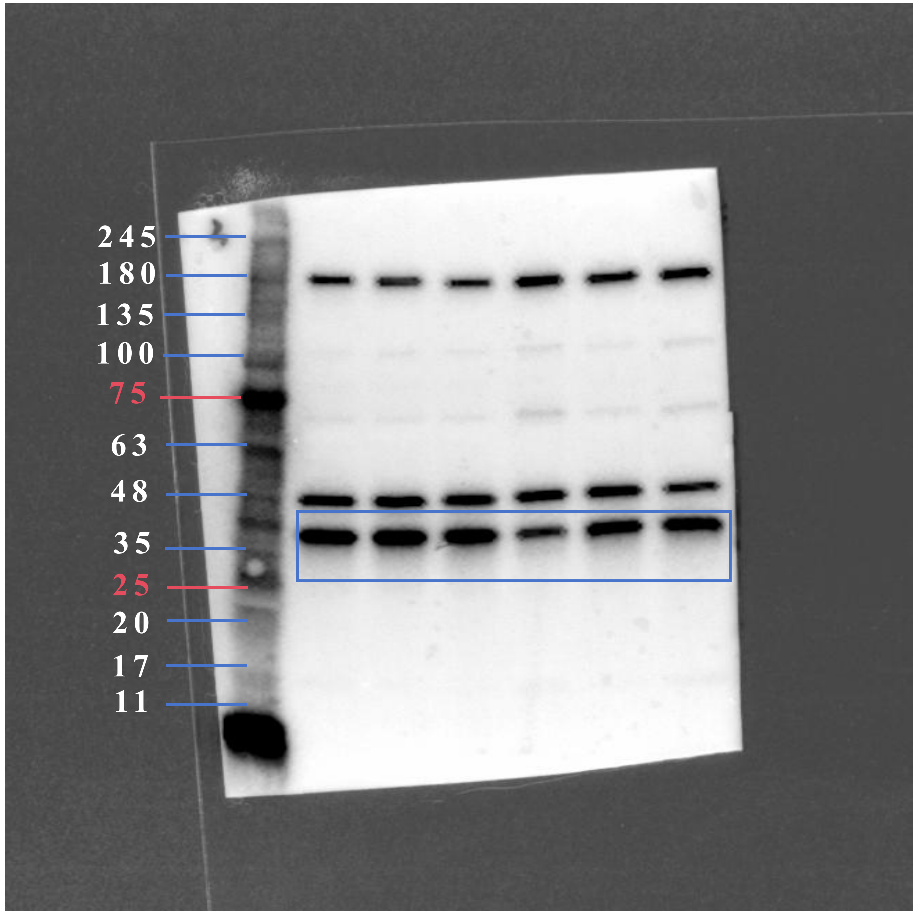

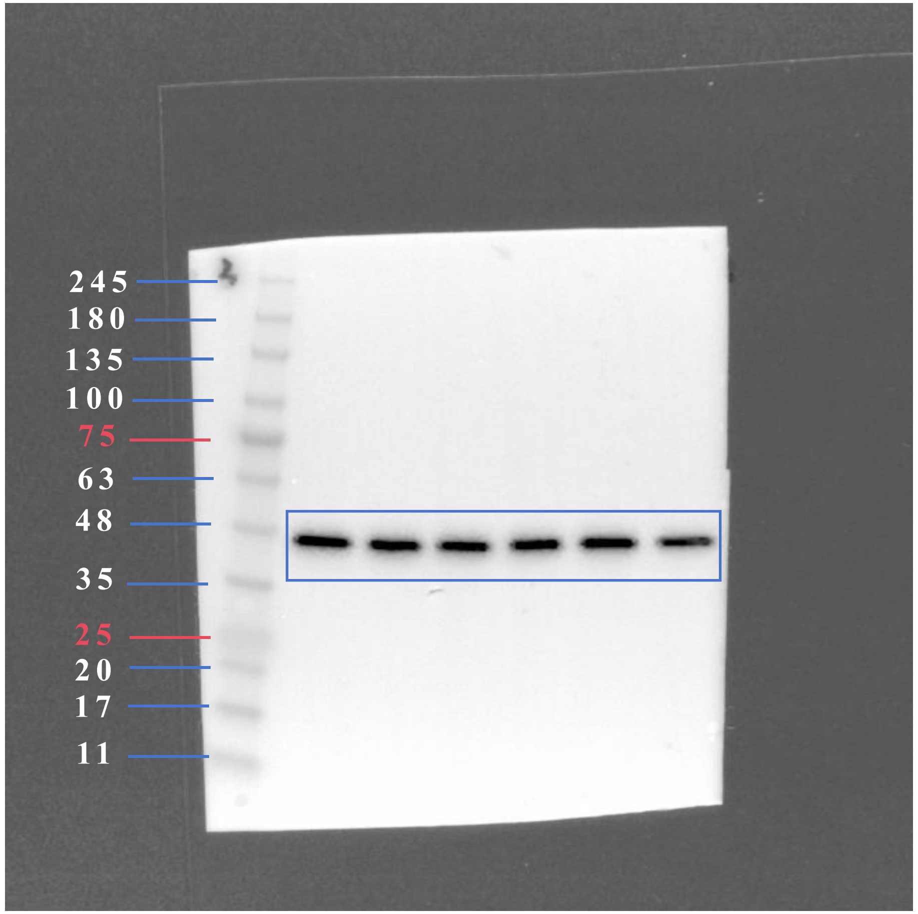

Lane1: Control

Lane2: BOT

Lane3: BAY11-7085

Lane4: TNF-α

Lane5: TNF-α+BOT

Lane6: TNF-α+BAY11-7085

Supplementary Figure S24.
Uncropped Western blot corresponding to Figure 9 (e) (ikb), Replicate 3. Target protein and β-actin were detected on the same membrane using the same protein lysates. The images show the full membranes from an independent biological replicate used for quantitative densitometric analysis presented in Figure 9 (e). All lanes are shown. No brightness or contrast adjustments were applied to individual bands.

Supplementary Figure S25. Three-dimensional HPLC chromatogram of the BOT extract

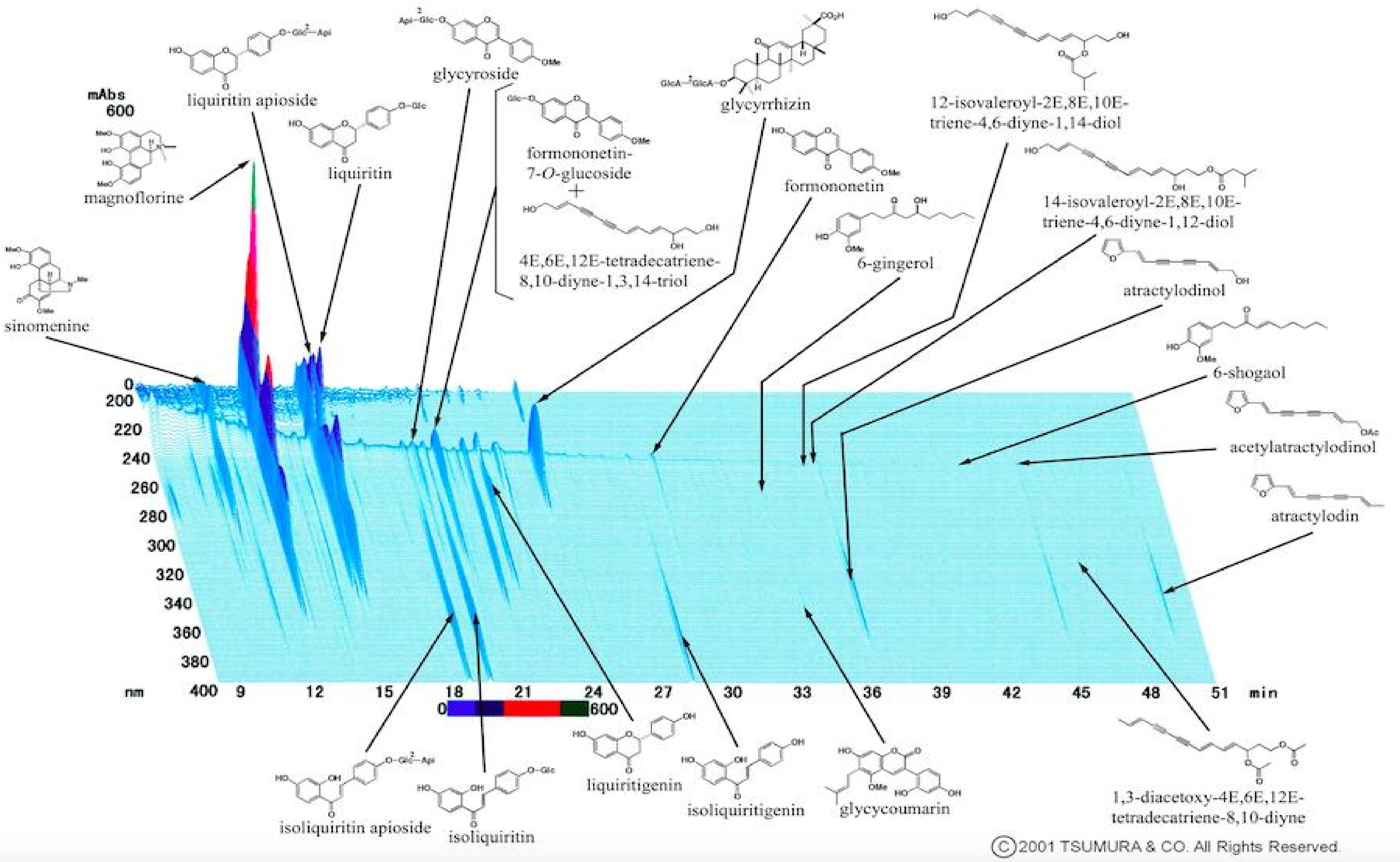

© 2001 TSUMURA & CO. All Rights Reserved.
